# Move-play-explore in early childhood education (MoveEarly) – study protocol of a cluster randomized controlled trial of staff professional development for quality pedagogical practices and holistic child development in Norwegian kindergartens

**DOI:** 10.1186/s12889-025-25831-1

**Published:** 2025-12-10

**Authors:** Eivind Aadland, Alicja Renata Sadownik, James Rudd, Hege Eikeland Tjomsland, Anthony D. Okely, Pernille Buschmann Eriksen, Maria Grindheim, Tracey Joyce, Kine Tveten, Thilde Kleppe Vårnes, Kjersti Johannessen, Katrine Nyvoll Aadland, Elin Eriksen Ødegaard

**Affiliations:** 1 Department of Sport, Food and Natural Sciences, Faculty of Education, Arts and Sports, Western Norway University of Applied Sciences, Campus Sogndal, Box 133, Sogndal, 6851 Norway; 2 Department of Pedagogy, Religion and Social Studies, Faculty of Education, Arts and Sports, Western Norway University of Applied Sciences, Bergen, Norway; 3 Department of Teacher Education and Outdoor Studies, Norwegian School of Sport Sciences, Oslo, Norway; 4 School of Social Sciences, Faculty of the Arts, Social Sciences and Humanities, University of Wollongong, Wollongong, Australia

**Keywords:** Preschool, Public health, Pedagogy, Continuing Education, Enriched environment, Child development, Physical activity, Motor skills, Creativity, Physical fitness, Self-regulation, Well-being, Integration

## Abstract

**Introduction:**

Physical inactivity in young children and schoolification within early childhood education are worrisome trends that curtail young children’s natural inclination to move, play, and explore. There is a need for high-quality evidence on effective, sustainable, and scalable interventions integrating the concepts of movement, play and exploration in early childhood education. Realization of such efforts depends on kindergarten contexts and requires highly competent staff. Through transdisciplinary intervention research, we seek to respond to these child development challenges by developing and testing a holistic move-play-explore early years pedagogy. The main aims of the MoveEarly study are to investigate the 1) effects of professional development for kindergarten staff on child movement, play and exploration and whole-child development and 2) how different kindergarten contexts influence implementation and institutionalization of the intervention.

**Methods:**

The study use a two-arm (intervention, control) cluster randomized design with 7- and 18-months follow-ups and will be conducted in Norway 2024–2026. We aim to recruit 50 kindergartens and 500 children aged 4 years to provide sufficient power to detect effect sizes between 0.25 and 0.40. The intervention is nested within two levels: the kindergarten and the child. The main intervention is an 18-month professional development/education module for kindergarten staff, aimed at supporting staff in developing their capacity to deliver an early years pedagogy integrating movement, play and exploration for all children. The outcome evaluation (4-year-olds) includes a range of child-level outcomes, including physical activity, physical fitness, movement competence, creativity, adiposity, socioemotional health, well-being, self-regulation, and early academic performance. The process evaluation (staff and all children) will describe implementation and adaptation processes using several types of quantitative and qualitative data.

**Discussion:**

Professional development of staff targeting a whole-child early years pedagogy that integrates movement, play and exploration in the kindergarten setting may provide a feasible avenue to enhance child health, development and learning. MoveEarly is designed to test this hypothesis to provide evidence for an early solution to lifelong public health and developmental challenges.

**Trial registration:**

www.ClinicalTrials.gov identifier NCT06488508 (05.07.2024).

**Supplementary Information:**

The online version contains supplementary material available at 10.1186/s12889-025-25831-1.

## Background

To lay the foundation for equitable development of human potential, life opportunities, well-being, and public health, providing every child the best start in life should be a top priority [1, 2]. Contemporary trends of “schoolification” within early childhood education (ECE) and high levels of physical inactivity in young children are two worrisome trends that are counterproductive to this aim [3, 4]. In this study, we will address these societal trends curtailing young children’s natural inclination to move, play, and explore. Through designing and testing an intervention to promote movement, play, and exploration in ECE, we respond to recommendations to support broader education goals and development of the whole child to equip them with the competencies they need to thrive and navigate a complex world [5, 6]. Given the extensive reach and the increased expenses allocated to ECE (in Europe) [7], this setting is optimally placed for developing quality practices favourably influencing health, development and learning of young children.

### The need for an integrated ECE pedagogy to support development of the whole child

MoveEarly will address two pressing societal challenges. First, the pressure to support the development of children’s cognitive and academic skills has led to a narrow understanding of young children’s development and an influx of inappropriate instructional practices in ECE [3, 8]. This trend, referred to as “schoolification”, is not supported by evidence and is likely to be detrimental to young children [3]. Causing much controversy and debate, schoolification is gathering momentum globally, including in the Nordic countries, curtailing children’s natural inclination to move, play and explore [9]. Consistent with traditional Nordic approaches, more nonlinear age- and play-responsive approaches that also take cultural historical perspectives into account are needed [10, 11]. Second, the global society is facing complex challenges with respect to inactive and sedentary lifestyles, which have their genesis in early childhood [12]. Physical activity (PA) plays a key role in preventing lifestyle-related diseases [13, 14] and emerging evidence suggest that PA beneficially affects brain health, cognition, and learning in children [15–18]. However, inactivity among children and youth, including kindergarteners, is high [4, 19]. Since many children spend most of their awake time in ECE, this setting is critical to provide children ample movement opportunities to ensure optimal health and development [7, 20, 21]. Previous ECE interventions to improve PA and related outcomes have lacked effectiveness and sustainability [22–26], requiring greater knowledge on integration of such interventions in ECE to shape policy. In response to these challenges, MoveEarly will pioneer transdisciplinary research integrating perspectives in ECE pedagogy, movement sciences, and public health with a focus on three interconnected pillars: *move, play, and explore*.

Movement is an inherently important part of young children’s being and becoming in the world [11]. Movement provides children with opportunities for play and exploration and promotes motor, cognitive, and social-emotional function and well-being [27–30]. International and Norwegian guidelines recommend children aged 1–5 years should move at least 180 min per day, of which at least 60 min should be of moderate to high intensity for children aged 3–5 years [31, 32]. Theories such as embodied cognition [33, 34]—supported by evidence in young children [35]—integrate the motor and cognitive domains, connecting movement with the mandate of ECE to promote both health and academic learning outcomes. Prevailing theories emphasize the bi-directional relationships between movement competence, motivation, and enjoyment during early childhood, and their importance for PA, as well as physical, mental, and social outcomes across the life span [36, 37]. Thus, movement represents a viable means for integrating public health and education during the early years. However, movement competence and physical development are generally lacking as ECE quality indicators [8, 38]. There is a need for pedagogical designs and outcome development that better promote and capture these important aspects of children’s behaviour and development.

Play is a core concept of ECE pedagogy, and its value for children’s development is undisputed. In play, children and adults co-create a shared imaginative world, where experiences can be adjusted with reality [39]. Ludic behaviour entails the quality of being joyful, energetic, and full of excitement, often seen in fantasy play and in gaming. Such ludic behaviour includes both movement and exploration [40]. Given that movement is a fundamental part of play in young children, movement is inherently a central aspect of play invitations and related learning outcomes. However, when focus on the educational or health benefits of play becomes too dominant, joy – an essential feature of play – is lost [41]. Children’s play is under pressure due to its use as an instrument to promote educational [3, 42] and health outcomes [43]. There is need for responsive pedagogical approaches to balance child-initiated and -directed play and exploration with the well-intentioned learning and health promotion objectives of ECE.

Exploration, an ubiquitous concept in ECE, is closely associated with curiosity, a natural disposition [44]. There is a fine line between ludic behaviour (play) and epistemic behaviour (as exploration and learning) [40] and children often switch between them. In explorative play, seriousness and concentration are often observed [40]. Through exploration, children experience and expand their capabilities in interaction with the world around them and learn to self-regulate and control their own thinking and behaviour. Exploration and movement are reciprocal functions, as perceptual information specifies the current constraints and opportunities for movement, while movement in turn generates perceptual information used to determine which actions are possible [45]. Though recently challenged by “schoolification” and inappropriate instructional approaches, shaped by assessment paradigms and “teaching to the test” [3], exploration is a common means of promoting early child development in *guided play* [42], *sustained shared thinking* [46], *discovery learning* [47], and *exploration as a dialogical engagement* [48]. From an ecological worldview on movement skill acquisition, learning occurs through a balance of unstructured and structured play through exploration, invention, and adaptation of possibilities for action in different contexts and environments [49]. Simply, what is ‘acquired’ is a functionally adaptable and evolving fit between the action capabilities of an individual and the constraints of the environment [50], emphasizing learning as a deep and practical knowledge developed by ‘doing’ [45, 51]. Thus, exploration is a common denominator in the fields of ECE and movement sciences, being central to a pedagogical approach to promote young children’s movement behaviours and holistic development.

### The need for co-creation of professional development in ECE

Studies of ECE interventions to increase PA [22, 24–26] and movement skills [23, 25, 26] have shown null or small-to-moderate effects. Specifically, effectiveness trials, which are conducted in real-world conditions, have generally not demonstrated success. At present, there are no successful, scalable strategies to increase PA in ECE settings, leaving policymakers and practitioners with limited evidence to guide their efforts. Great effort and thus competent and motivated staff are required to promote and integrate PA in an enjoyable way with other kindergarten routines, activities, and objectives. However, most studies promoting PA and motor skills in ECE have provided minimal professional development and support [26], while long-term, intensive training probably is needed for such comprehensive interventions [52]. Some studies have provided more teacher training, workshops, and support and established stronger relationships between interventionists and staff [53–57], however, still generally showing equivocal effects.

The lack of staff training in many studies is concerning because low staff competence in the kindergarten sector internationally and in Norway is a challenge for program implementation [7], in particular for promoting PA and motor skills. This calls for development and implementation of quality professional development programs to support intervention implementation [58]. Little is known, however, about the effects of professional development in ECE and few evaluated professional development programs are available for the ECE workforce [26, 52]. While face-to-face teacher training is the most common approach [25, 26], a recent focus has been the provision of online PA teacher training as these approaches may provide higher levels of compliance and broader intervention reach at a lower cost [59–64]. Such studies have shown favourable effects on teacher outcomes [62, 63], but less is known about effects on child outcomes [60, 64]. Finally, given the need to tailor interventions to kindergarten staff [58, 65], their views on barriers and facilitators of various training approaches and how these approaches potentially could be optimally integrated to fit their needs, is important.

Hawe et al. [66] argue that public health interventions traditionally have focused over-simplistically on the “package” of activities delivered rather than on the dynamic and complex context in which the intervention is introduced. Since kindergartens are complex ecological systems, developing staff competences and providing resources might not be effective without taking the complex social system where staff are situated into account [66–68]. Thus, kindergarten interventions need a systems-level approach to understand how the intervention (hypothetically) integrates with or interrupts the prevailing practices and might cause change in practice. As each kindergarten has its own social system and contextual factors that need to be considered when delivering the intervention, flexibility and adaptability are key when interventionists approach kindergartens [24, 58, 65]. In contrast to strict and standardized interventions, interventions could be tailored by standardizing function, rather than form [66, 69]. To better understand kindergartens’ social systems and staff’s roles, relationships, and needs, interventions should be co-created with researchers, users, and stakeholders to bring rationales, success factors, and barriers – as experienced by all parties – to the table [70, 71]. In addition to allowing all parties to buy in, a thorough process of co-creation allows a collective understanding of the system, identifying opportunities for interrupting it, and shaping an intervention’s theory of change [72]. A well-developed theory of change is crucial for shaping a good intervention and for designing and conducting an informed process evaluation [68, 73, 74].

### The need for broader evaluation

ECE assessment regimes of early learning, for example, as applied by the Organisation for Economic Cooperation and Development [38], do not sufficiently capture children’s holistic development. Broader measures of child development, including physical development, movement competence, creativity, playfulness, explorative behaviours, and well-being, are needed to evaluate interventions and to guide ECE policy and practice. However, existing assessment tools of motor skills set their scale of analysis exclusively within the child and have little appreciation for the environment or context [49, 75]. Little research has targeted movement competence measures in young children from an ecological and dynamic perspective, which take the embodied and embedded nature of movement into account [49]. Moreover, evidence on the relationship between PA and subjective well-being in young children is lacking [29], largely due to a dearth of appropriate child-report measures in this age group. Beyond such outcome assessments, qualitative approaches are needed to obtain a deeper understanding on children’s perceptions as quantitative approaches cannot fully capture the experiences or the learning affordances children are provided [76]. Finally, because randomized controlled trials (RCTs) are not primarily focused on explaining why an intervention may work in a particular context, or for a particular group of participants, examining how an intervention is put into practice is critical to inform scaling to the real world [68, 73, 77].

### Objectives and research questions

Connecting and developing ideas and concepts across disciplinary boundaries is a prerequisite to developing quality solutions to complex societal challenges. In response to the need for an integrated perspective on the role of movement, play, and exploration in young children, we will design and test an integrated early years pedagogy to provide a synthesis of contemporary perspectives across disciplines and to stimulate new transdisciplinary theoretical, methodological, experimental, and empirical research in the ECE field. We propose MoveEarly as an efficient, acceptable, and feasible solution to child development challenges and to shift societies toward a more active, flourishing, and sustainable way of life. In response to low staff qualifications, we will raise competence and facilitate implementation of the move-play-explore pedagogy by offering ECE staff a quality professional development program structured within a continuing education model. An online resource will be an essential part of this program, supporting implementation of MoveEarly and later scaling. To understand kindergarten contexts and staff needs and to allow all parties to buy in, the intervention will be co-created between researchers, users, and stakeholders. This is crucial for designing both a good intervention and an informed process evaluation.

In response to the need for broader evaluation of interventions in young children, we will develop and use new outcomes of movement competence, creativity and well-being. We will use qualitative approaches to obtain a deeper understanding of children’s perceptions and particularly how exploration and interaction with their physical and social environment facilitate and consolidate learning. Beyond outcome evaluation, we will conduct a thorough process evaluation to explain discrepancies between expected and observed outcomes (on an individual and organizational level) to better understand how context influences outcomes. By identifying the mechanisms of implementation, we will inform scaling of the intervention in the real world.

The main objectives of MoveEarly are to develop and test:

- a novel ECE pedagogical approach integrating movement, play, and exploration as conceptual cornerstones for improved child development
- a novel professional development and resources for the ECE workforce to implement movement, play, and exploration in their everyday practice

The primary research questions that will be investigated in MoveEarly are:

1. How does the MoveEarly intervention impact children’s PA, physical fitness, movement competence, creativity, playfulness, explorative behaviour, social-emotional health, well-being, self-regulation, and early academic learning?
2. How does the MoveEarly intervention interact with different kindergarten contexts to produce various individual and organizational outcomes?

The secondary research questions that will be investigated in MoveEarly are:

3) How does “move, play, and explore” align with fundamental concepts of ECE pedagogy and how will these concepts inform the development of a responsive pedagogical design?
4) How do historical and cultural conditions of kindergartens enable move-play-explore integration in ECE?
5) Which measurement properties do the developed assessment instruments of movement competence, creativity, playfulness, explorative behaviour and well-being have in young children?
6) How can co-creation of professional development and kindergartens’ development work inform and shape sustainable solutions and quality resources for the kindergartens?

## Design, timeline and intervention model

### Study design

The MoveEarly study will use a cluster RCT design with randomization at the kindergarten level, including a mixed-methods approach [78]. This protocol follows the SPIRIT Statement [79] (Supplemental Table 1) with extension of the intervention description according to the TIDieR checklist [80]. Reporting of the RCT will follow the CONSORT Statement with extensions for cluster trials [81] and pragmatic trials [82]. The trial is registered with an international trials registry prior to recruiting participants (registry https://www.clinicaltrials.gov; identifier NCT06488508; date 05.07.2024).

**Fig. 1:**
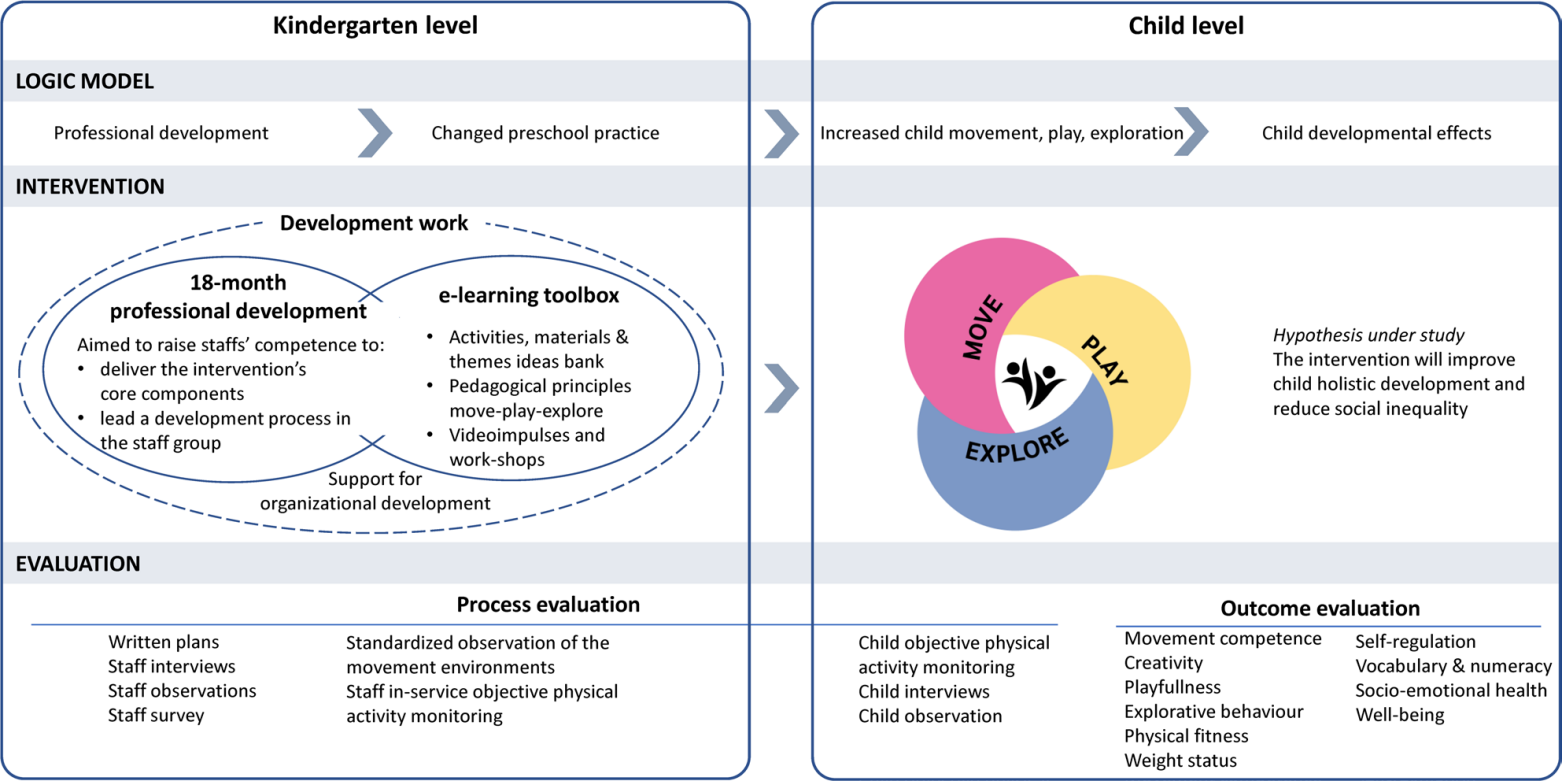
A simplified intervention model of the MoveEarly cluster RCT study

The intervention will be co-created with kindergarten owners and staff to provide broad support and anchor the project in the kindergarten sector [71]. By allowing each kindergarten’s contextual factors to be reflected within the larger cluster RCT [65], we aim to increase the value for subsequent scaling and overcome a common criticism of clinical trials. To further support scaling, the study is framed within a “realist RCT” approach [73], where a realist evaluation is nested within the RCT [83]. As shown in Fig. 1, the intervention children receive is aimed at increasing their movement, play, and exploration during an 18-month period (November 2024–June 2026). To support staff in promoting movement, play, and exploration among children, kindergarten staff will be offered a professional development program/education module *MoveEarly* (optional 15 credits) including an online resource that will provide on-going, concrete support over 18 months. The intervention will, depending on each kindergarten’s current practice, add to, extend, and/or integrate with and improve existing activities [84]. The control group kindergartens will continue their normal practices and may receive the intervention after the trial is completed.

### Timeline, randomization and blinding

**Fig. 2:**
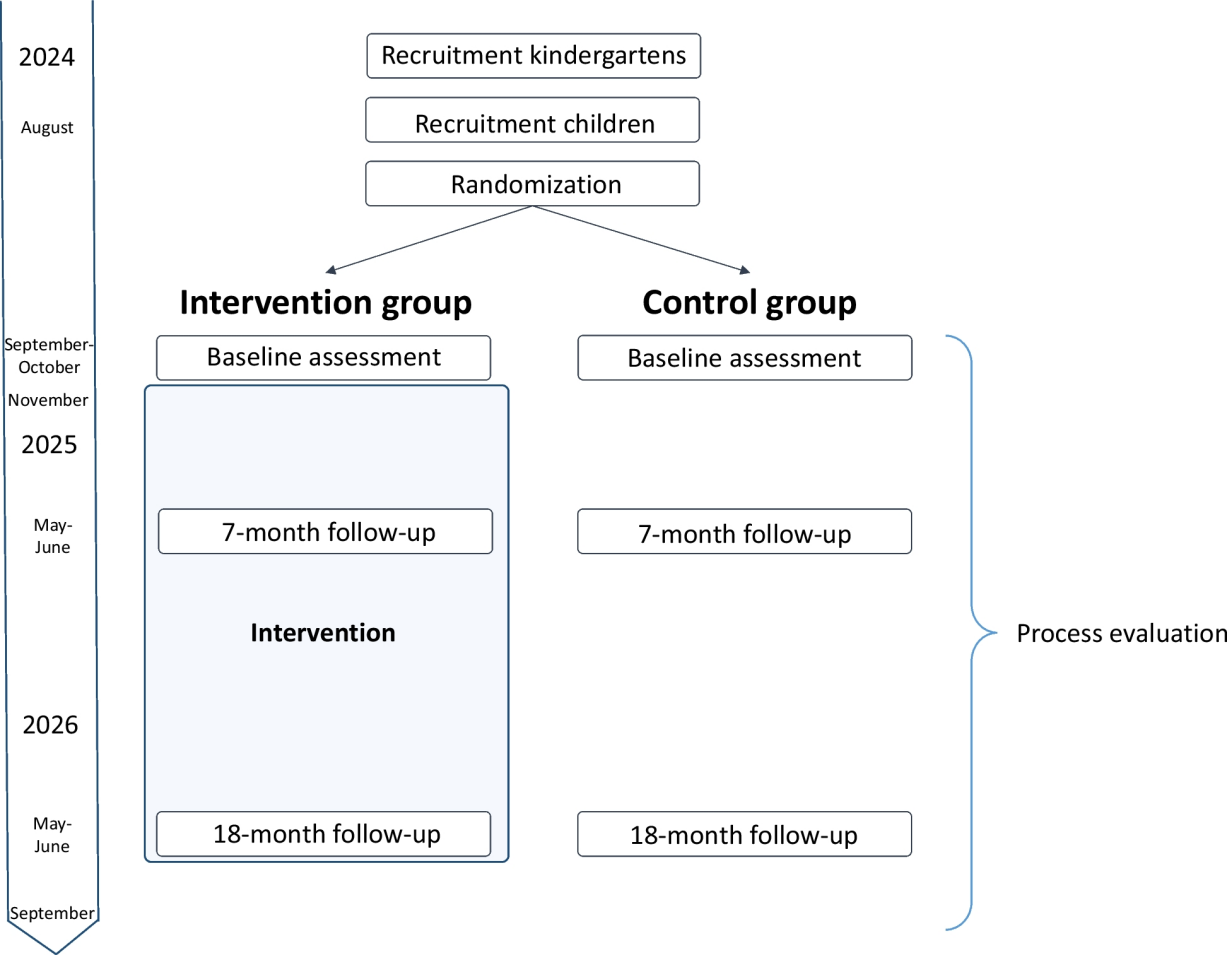
Flow chart and timeline of the MoveEarly cluster RCT study

Figure 2 shows the flow chart of the intervention. Collaboration with kindergarten owners was initiated in 2020, before applying for funding. Kindergartens that were candidates for participation (i.e., all kindergartens of the participating owners) were informed about the project development on several occasions between 2022 and 2024. Kindergartens will be recruited in May 2024, while children will be recruited in August 2024. Baseline data collection will be performed September–October 2024, before the intervention start in November 2024. Staff will receive an 18-month professional development with a short-term follow-up (7 months) on selected outcomes and a long-term follow-up (18 months) on all outcomes to allow a comprehensive outcome evaluation. Process evaluation is ongoing during the intervention period.

We will cluster randomize participants at the center level since we regard delivery of the intervention on the center level the only practical approach to avoid contamination. An independent researcher will randomize 50 kindergartens to the intervention group (25 kindergartens) or the standard of care control group (25 kindergartens), with stratification on the two kindergarten owners. Randomization will be performed using a simple random number generator (SPSS) and a 1:1 ratio for the 2 intervention arms when the list of participating kindergartens, sorted alphabetically for each owner, is complete. The list of centers allocated to each group will be handled by the primary investigator. The primary investigator will inform kindergarten directors about their group after completing recruitment of children. Blinding of participants and kindergartens is not possible due to the nature of the experiment. However, we expect that children will not be aware of their group assignment. To limit potential biases, the fewest possible researchers and assessors involved in project management, data collection, and statistical analysis will have knowledge about the group assignment and the study hypotheses.

### Intervention and theoretical framework

The MoveEarly intervention comprises several causal assumptions related to the complex societal problems addressed in the project. These assumptions are visualized by the program theory presented in Fig. 1, presenting the inputs of the intervention as well as the processes and mechanisms possibly evoked by the intervention targeted to produce change at the kindergarten and child level. In line with a socioecological model [85], the main assumption underpinning the intervention is that when researchers work in partnership with kindergarten staff to enhance their competence and motivation to integrate the move-play-explore pedagogy into their daily practices and the staff change their practices, this will positively impact children’s developmental outcomes. Common across kindergartens will be the flexibility to tailor the intervention to ensure staff engagement and to meet the contextual needs and constraints of the kindergartens [65]. We will therefore not specify the precise form the intervention might take in each kindergarten but will specify key intervention principles and components. How intervention implementation and adaptation vary according to individual and contextual factors, will be examined in the process evaluation.

Beyond key information about the intervention presented below, more details about the approaches taken to develop the interventions are provided in Sects. " Conceptual groundwork for design of the move-play-explore pedagogy (child level)" (child level) and Co-creation of a continuing professional development program and intervention delivery (kindergarten level).

#### Child level – A move-play-explore pedagogy

**Fig. 3:**
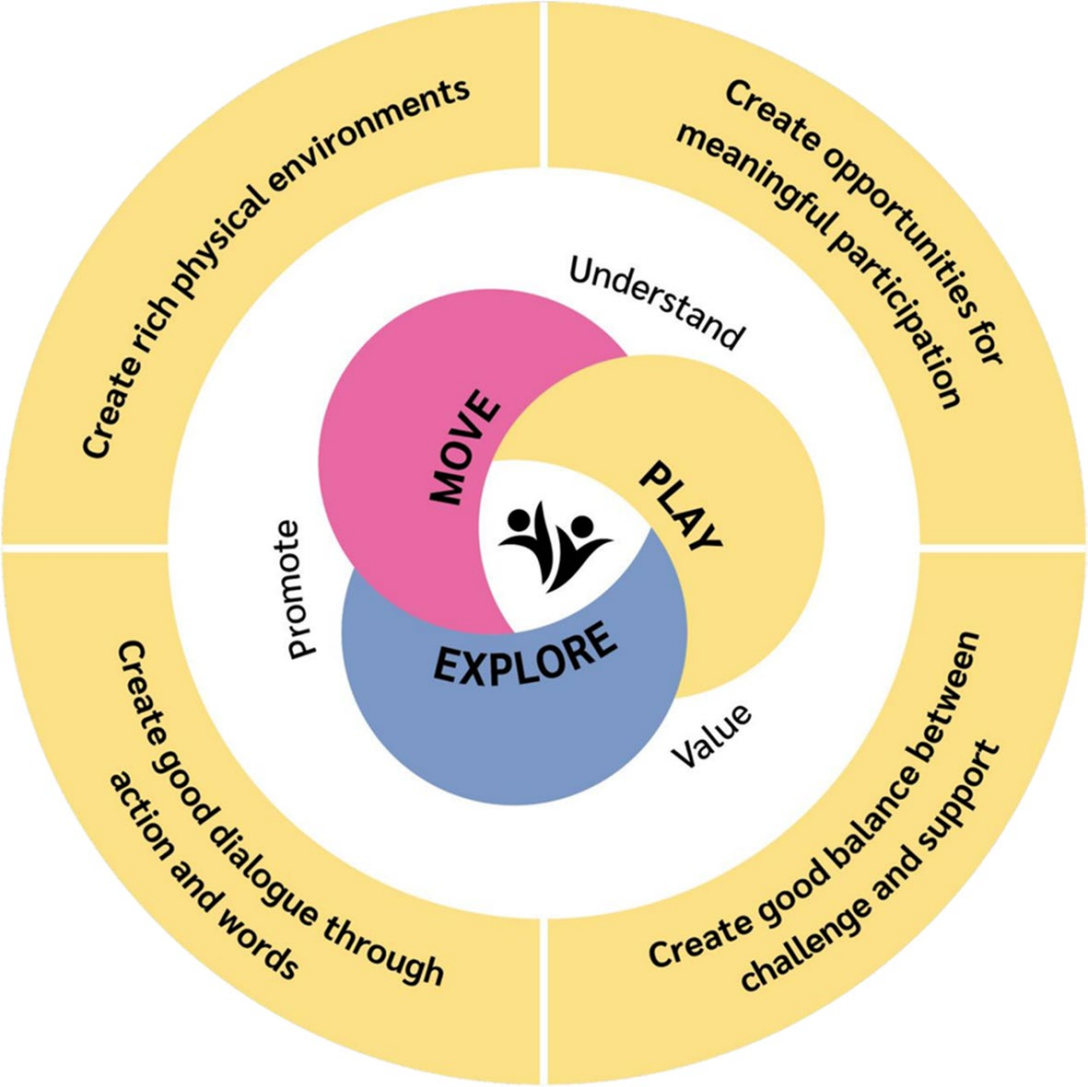
The pedagogical principles underpinning the move-play-explore pedagogy

The child level intervention is framed within seven pedagogical principles which staff is supposed to implement over the 18-month intervention period (Fig. 3). The theoretical framework underpinning these principles will be transdisciplinary, integrating collaborative knowledge building and dialogical interaction with context (cultural-historical) and environmental (ecological) approaches [27, 33, 46, 48, 49, 86]. There will be published a conceptual paper; however, a description of the principles is provided here. The core of the intervention is to value, understand and promote movement, play and exploration. Staff may achieve this goal by developing quality practices including creating rich physical environments and creating quality staff-child interactions characterized by balancing challenge and support, explorative dialogues and providing children opportunities for meaningful participation. The intervention is not a programme prescribing specific schedules, teaching approaches, activities or intervention dosages, durations or intensities, but principles guiding the adoption of a holistic early years pedagogical approach tailored to kindergartens’ contexts and children’s needs and interests. Staff are flexible with respect to how (e.g., individual children, small groups, larger groups), when (e.g., time of day, duration, number of sessions) and where (e.g., indoor, playground, nearby area outside of kindergarten) the principles are implemented. The principles may expand, extend and/or integrate with existing practices and activities [84].

##### Understand, value and promote movement

Children sense and experience through their whole being. As such, sensing and moving is fundamental for young children´s way of navigating the world around them. They understand themselves, others and their surroundings through feeling, touching, observing, mirroring, trying, negotiating and interacting using their bodies. Staff in ECE can meet children´s natural inclination for movement, enrich their experiences and provide a strong basis for lifelong, positive activity habits by integrating movement in everyday activities. This can be done by:

- Integrating movement and bodily experiences in all subject areas (i.e., curriculum)
- Arranging for a broad spectrum of motor and sensory experiences in play and exploration
- Offering varied activities that requires high intensity, but also ensure sufficient rest
- Promoting activities challenging children’s motor skills, self-regulation and/or social competence
- Responding with engagement and enthusiasm on children´s actions and experiences
- Allowing space for small and big movements, as well as bodily expressions

##### Understand, value and promote play

Play is a way of being for children. Play is a surplus phenomenon and a source of experiences and development of knowledge and competencies. The main characteristic of play is imagination. Play can be a way of challenging understanding and norms and requires the ability to see new ways of using materials, surroundings and yourself. Play can result from exiting as well as frightening experiences, or simply from energy surplus. The ECE must make play accessible for all children. This can be done by:

- Recognizing and acknowledging the different expressions of play for children and oneself
- Being conscious of the continuum of play (from free to structured play)
- Continuously reflecting on one’s own role in children´s play and how to get involved in play
- Protecting, facilitating for and expanding children´s initiatives, imagination and play
- Ensure both children and staff have opportunity, time, and energy to play
- Be creative together with the children and use materials in a variety of ways

##### Understand, value and promote exploration

Children navigate the world they live in through exploration of the unknown. When a child persists in a task, and has supporting conditions for this, the unknown becomes known. Thus, by trial and error, they develop new understanding and knowledge. Moreover, when the unknown becomes more known, the opportunity for play opens up. Hence, staff must value the child’s exploration, trial and error. This can be done by:

- Understanding that children gain control of the unknown by exploring and learn new skills by trial and error
- Facilitating for a variety of experiences by letting children meet and try new materials, landscapes, cultures, activities, and situations
- Giving children room (time and space) for experimenting and exploring
- Promoting activities with open solutions
- Valuing a variety of approaches and creativity in activities and solutions
- Valuing process over product through acknowledging, praising and commenting on effort and engagement in trial and error

##### Create rich physical environments

Children’s development and well-being are associated with stimulating outdoor and indoor environments that invite movement, play and exploration. Hence, it is important to create environments that promote a variety of interactions between children and their surroundings, including the physical environment, other children and staff. This affords children a broader spectrum of ways of being, thinking and acting, which will form their meaning making and life. Staff can do this by:

- Offering variation in surroundings, surfaces, and material to inspire and challenge children outdoors and indoors
- (Re)arranging furniture to give room for a variety of movement, play and exploration
- Spending time outdoors and using the variation different seasons, weather and nature creates
- Supporting children´s initiatives and expanding their ongoing activities by offering relevant materials
- Organising the child group in appropriate ways
- Creating possibilities for children to explore and express themselves on their own terms and in their own tempo, together with other children and with staff

##### Create opportunities for meaningful participation

Children´s motivation, engagement and development have best conditions when they experience meaning and meaningful participation. For children to experience the joy of moving, playing and exploring to a greatest possible extent, it is important to understand what motivates a child. This can be done by:

- Adapting activities for all children based on their individual premises and interests
- Letting children co-create activities
- Following up on children´s interests, initiatives and curiosity in ways that are meaningful for others
- Supporting children´s participation by guiding them or joining play or other activities
- Facilitating for a variety of physical environments where the children can make choices
- Offering activities and environments that make children explore and use materials in creative ways

##### Create good balance between challenge and support

A good balance between challenge and support is important for children´s optimal development. By achieving this, the child will expand their experiences of having to challenge them self and to master a particular skill or task. In safe conditions, children can push their limits, dare to try something new, and take risks. Staff can support this by:

- Facilitating for a variety of activities that ensures that children experience challenge and mastering
- Being conscious about when to support and help, and when to let children try themselves
- Guiding and being close to children when they challenge themselves in difficult activities and risky play
- Adapting environments, activities and materials to let every child experience progression and development
- Enriching and expanding activities initiated by the children
- Inspiring children to try new activities and new materials

##### Create good dialogue through action and words

Dialogue and communication can enrich children´s understandings, meaning making and ability to think in new ways. Children explore and co-create meaning through verbal and non-verbal dialogue with adults, other children and their surroundings. Hence, staff must create room for enriching dialogues. This can be done by:

- Participating in activities and modelling movement, play and exploration
- Asking open questions that invite conversation about new and known subjects
- Exploring concepts with children through talking and doing
- Facilitating dialogue between children so that they can collaborate in problem solving activities
- Talking with children and inviting reflection around how they use their bodies and what they experience when they move, play and explore
- Suggesting activities for children who find it difficult to play, who often choose sedentary activities, or who wander around

#### Kindergarten level – professional development and resources

Our overarching approach to professional development is rooted in Vygotsky’s sociocultural theory [87]. We acknowledge mutual respect, trust and growth and aim to establish strong partnerships between kindergarten staff and researchers where both parties can share knowledge and expertise and co-create the MoveEarly-pedagogy. Consistent with our flexible and pragmatic intervention approach [65], we regard constructive interactions with staff over the long term and tailoring of support to staff’s premises, needs, and the social contexts they are situated in central tenets underpinning staff’s development and learning. We understand staff as competent members of professional learning communities, for which we aim to promote cultures of collaboration by facilitating good leadership, functional group interactions, trust and respect [88].

Through the professional development, we will seek to create an autonomy-, mastery- and relatedness-supportive learning climate guided by Self-Determination Theory (SDT) [89, 90]. According to SDT, this learning climate will provide social-contextual conditions that help stimulate intrinsic motivation, self-regulation, and well-being through supporting the three basic psychological needs autonomy, competence, and relatedness. To achieve this, 1) we will allow and expect staff to set their own goals and make their own decisions, thus giving opportunities to develop initiative, independence, ownership and responsibility, 2) we will provide appropriate challenges and support, scaffold and structure to aid staff’s sense of success, and 3) we will show interest, respect and empathy for their challenges, processes and solutions. If the intervention succeeds in supporting basic psychological needs aligning with SDT, it is expected that staff may enhance their intrinsic motivation for learning, creativity, performance, self-esteem and enjoyment and thus improve their persistence and sustainability of the intervention. In turn, we anticipate these effects will affect children’s conditions for development, learning and well-being favourably [90].

Before starting the intervention, each kindergarten will decide on a suitable internal project group, constituted by the director and several teachers (typically one teacher from each department), who have the responsibility of leading the implementation of the project (i.e., the pedagogical principles) in each kindergarten. With a few exceptions where all staff members take part in the professional development (i.e., the second day of the first seminar and the kindergarten visits; see below), the professional development is mainly targeted towards the project group. They will be responsible for planning and implementing the intervention in their department/kindergarten, tailored to their contextual factors.

**Fig. 4:**
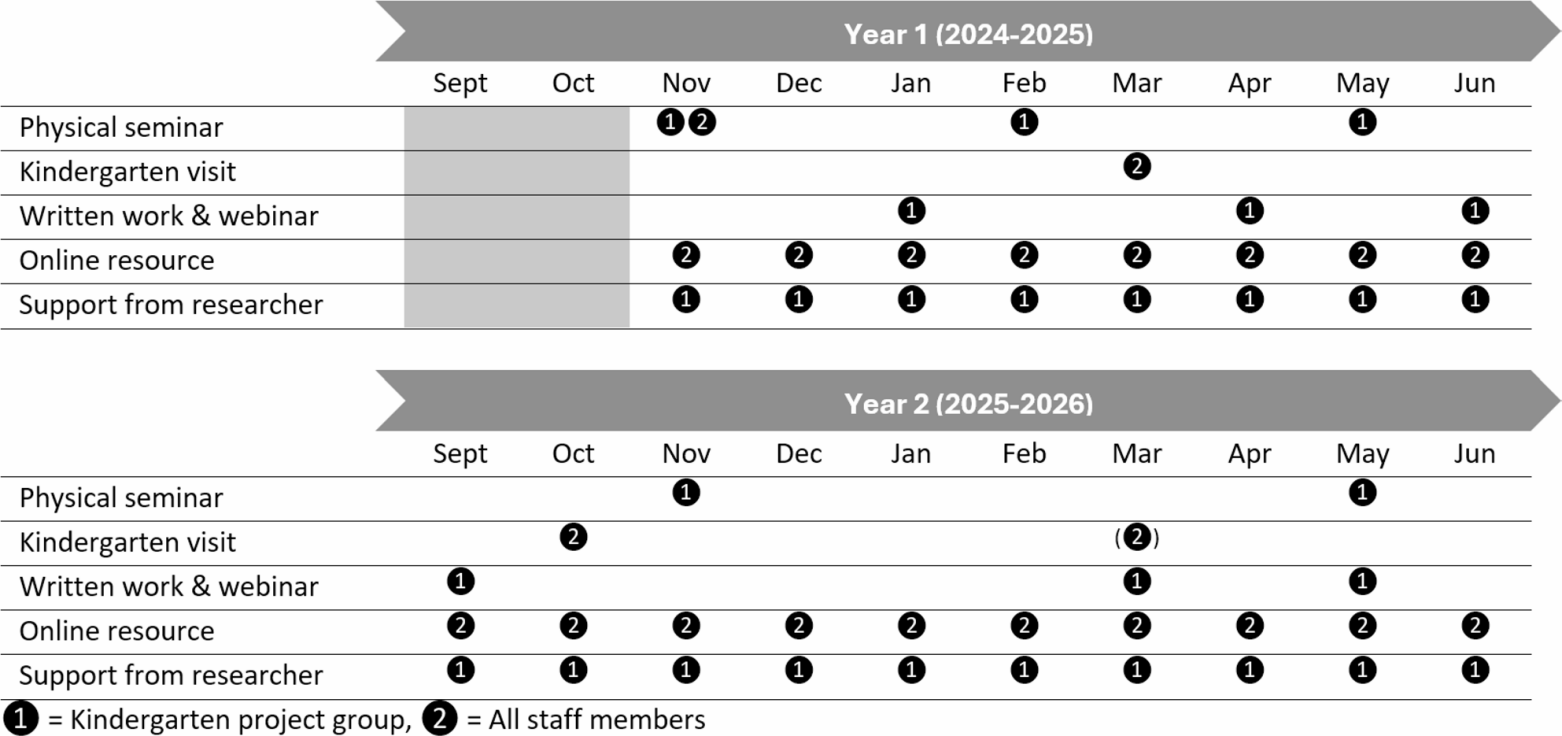
The design and timeline of the kindergarten level intervention

Together with kindergartens, we have designed an 18-month supporting process and resources for kindergartens on movement, play and exploration constituting 5 parts (Fig. 4) (see Sect. "Co-creation of a continuing professional development program and intervention delivery" for more details on this process).

##### Physical seminars

We will run 6 full-day physical seminars for kindergarten staff at the University campus. The intervention starts with a two-day seminar in November 2024. The first day will target the internal project groups and the second day will target all staff members in each kindergarten. Thereafter, there will be two seminars during Spring 2025, one seminar during Autumn 2025 and one seminar during Spring 2026. The seminars will include a mix of short lectures, practical sessions and workshops on the pedagogical principles and how to organize and support development work in kindergartens, as well as experience sharing among kindergartens.

##### Kindergarten visits

We will organize one physical seminar in each kindergarten during Spring 2025, one seminar during Autumn 2025 and one optional seminar during Spring 2026. The target group of these seminars is all staff members. The goals of these seminars are for researchers to observe each kindergarten’s practices (one half day) and discuss specific challenges staff face (1–3-h staff meetings). The kindergarten project group will lead these seminars and researchers will contribute with short lectures, practical sessions or workshops as requested by the project group.

##### Written work, including digital meetings

The project group will develop three different types of written work over the intervention period: 1) a project description, 2) a reflection paper, and 3) a project report. There will be one cycle the first year and one cycle the second year, meaning that all written works will be completed twice, with opportunities for adaptation and further development the second year based on visions, experiences, and turnover of staff members. The project description should include specific goals, milestones and action points for how the department/kindergarten will implement the pedagogical principles. The reflection paper may use various tools or approaches (storytelling, pictures, videos etc.) for reflecting on how or to what extent the pedagogical practices are consistent with the pedagogical principles. The project report should sum up any experiences on implementing the pedagogical principles and organizing the project and provide action points for future improvement. After hand-in, all written work will be subject of digital meetings with small groups (typically 2–4) of kindergartens for sharing of experiences and feedback.

##### An online resource (https://moveearly.no/, password required until the project is completed)

Continuing support will be provided by a project web page including three different parts: 1) An ideas bank of various movement, play and exploration activities, 2) an overview of the pedagogical principles, and 3) brief “videoimpulses” (i.e., 3‒10 min lectures) and workshops on the pedagogical principles and how to plan and organize development work in kindergarten. These resources are designed to complement the physical seminars and support both the project group and the rest of the staff members in their efforts to implement the move-play-explore pedagogy in their kindergartens. The resource is designed to work on computers, tablets and smartphones as a concrete 24‒7 resource over the intervention period.

##### Support from researcher

All kindergartens will have one researcher they may contact at any time with any queries regarding the move-play-explore pedagogy, implementation process, professional development, timeline etc. This researcher is the same person who will visit the kindergarten, thus having good knowledge about each kindergarten’s context and process, which may facilitate mutual trust and a stronger relationship. Frequency of contact is not predetermined but will be logged.

The specific content of each part will be tailored to fit kindergartens’ needs and will be logged and specified after the intervention is completed. The professional development will be led by researchers with expertise in physical activity, motor learning, public health, and/or early years pedagogy, all of whom have substantial experience working in or with kindergartens and/or kindergarten teacher education.

## Sample, sample size calculations and recruitment

The intervention will target all children in kindergartens irrespective of age. The target group of the outcome evaluation is 4-year-olds (at baseline) since: i) both evidence and experience indicate that obtaining reliable and valid assessments are very challenging in younger children, ii) this age group allow for 18-month follow up. The target group of the process evaluation will be kindergartens, staff and all children in the included kindergartens (ages 1‒5). We performed sample size calculations for the outcome evaluation based on standardized effect sizes reported in meta-analyses of interventions delivered by kindergarten staff investigating young children’s device-measured PA (0.27) [22] and motor skills (0.41) [23], and schoolchildren’s cognition (0.11‒0.30) [91] and academic achievement (0.16‒0.26) [92]. Derived from a conservative sample size calculation, including correction for the cluster RCT design, we aimed to recruit 50 kindergartens and 500 children to the study. This sample size allows detecting statistical significant standardized effect sizes (Cohen’s d) of 0.25‒0.40, given ɑ = 0.05, 1-β = 80%, group ratio 1:1, correlation of repeated measurements = 0.60‒0.70, intra-class correlation (ICC) = 0.05‒0.10, children per cluster = 9 after accounting for attrition and coefficient of variation for cluster sample size = 0.65 [93] (design effect correction factor = 1.60‒2.19). The assumed ICCs were derived from previous studies [20, 22].

The kindergarten inclusion criterion is having ≥ 6 children aged 4 years enrolled (i.e., born 2020); exclusion criteria are factors making it extraordinary difficult to implement the intervention (i.e., taking part in other extensive research- or development projects, having a very challenging workforce situation or having larger restoration or renewal work ongoing). All 4-year-olds enrolled in the included kindergartens will be invited to take part in the outcome evaluation. We will also invite all staff members in these kindergartens and all children of any age in a smaller sample of 12 case-study kindergartens to take part in the process evaluation. We will establish agreements with kindergarten owners on recruitment of kindergartens to ensure recruitment of a sufficient number of kindergartens. Recruitment of children will be carried out by the kindergarten staff. We will establish strong collaboration with kindergartens to ensure a good recruitment process and follow up kindergartens where recruitment is challenging. Written informed consents for children will be provided in several languages and interpretation services will be used as needed to meet parents’/guardians’ needs for information.

## Materials, methods, and approaches

### Development of intervention approaches

The two levels of the MoveEarly intervention (i.e., 1) the child level: the move-play-explore pedagogy; 2) the kindergarten level: the staff professional development and support) were co-created with kindergartens. The sections below briefly describe our approaches.

#### Conceptual groundwork for design of the move-play-explore pedagogy (child level)

##### Background

In this work, we examined how the core concepts of movement, play, and exploration defined their meanings and how they can inform a responsive pedagogical design—understood here as child-responsive practice—for the adoption of a holistic development approach in ECE. Conceptual groundwork is fundamental for generating new knowledge in transdisciplinary research [94]. The MoveEarly project involves three main research disciplines: ECE pedagogy, public health, and movement science. These fields actively use the concepts of movement, play, and exploration within the context of child development research. However, it is currently unknown how the definitions and descriptions of these concepts align across these discourses or how they overlap and interrelate within each discourse. Another key aspect of the conceptual groundwork is to gain clear insights into how movement, play, and exploration both occur and are perceived in Norwegian ECE settings. Developing a precise and coherent narrative about movement, play, and exploration is crucial to enhance the understanding and trust necessary for co-creating a pedagogical design centred around these concepts. Such a design must be responsive and attentive to the needs of all children within ECE.

##### Design and methods

We employed a combination of inductive and deductive approaches to gain a comprehensive understanding of the move-play-explore nexus for the MoveEarly project. The term "nexus" is pivotal as it signifies our aim to transcend siloed research, discourses and practices by embracing a transdisciplinary approach. Transdisciplinarity in the MoveEarly project transcends traditional disciplinary boundaries of ECE pedagogy, public health, movement sciences, and current kindergarten practices in Norway to create new forms of knowledge that are irreducible to the initial disciplinary components but have shared understanding and acceptance from the three disciplines involved [95].

###### Deductive approach

A scoping literature review will be carried out to identify how the concepts of movement, play and exploration are connected in academic literature concerning young children aged 1–6 years in ECE (10.17605/OSF.IO/MR75K). The search will be undertaken in two steps: 1) a systematic search in the databases Education Resources Information Center, Medline, Sportdiscus, Web of Science, Nordic Base of Early Childhood Education and Care, and 2) a hand-search in the journals Journal of Motor Learning and Development, International Journal of Behavioural Nutrition and Physical Activity and European Early Childhood Education Research Journal. Relevant articles published 2018–2024 will be included. This search strategy ensures a comprehensive review of relevant literature to understand how movement, play, and exploration are defined and understood across discourses, furthering our knowledge of synergies and differences across approaches to movement, play and exploration.

###### Inductive approach

The Norwegian Framework Plan for Kindergartens emphasizes the importance of movement, play, and exploration in ECE [96]. Emerging research on play [10, 97–100], outdoor activity [101–103], and exploration [104–107] underscores the need to investigate how these elements are embodied in daily kindergarten practice. The inductive approach provides insights into the situated and declared understanding of the move-play-explore nexus in kindergartens. 

To study situated conceptualisations of movement, play, and exploration, we will engage kindergarten staff and children in “guided tours”, a participatory research technique where participants guide researchers through familiar spaces, providing deep insights into their embodied and situated knowledge [108, 109]. ECE staff and children are positioned in the roles of experts, who guide the researchers (visitors) around a space that is familiar and professionally or personally meaningful for them. Such positioning gives the researchers possibility to access deeply situated and embodied knowledge, mediated through the stories, actions and body language of the research participants. Video and audio recording these visits will allow for documentation of the verbal and nonverbal storytelling on the “local ecologies” [110], revealing the way the participants interact with and experience the space, and what meanings they attach to different areas. After the tour, focus groups with the involved kindergarten staff will be used to study the staff’s reflection over the places/spaces, activities and understanding that appear and become visual/verbal during the guided tours. Guided tours will be complemented by staff’s spontaneous filming of instances of children's activities interpreted as involving movement, play, and exploration to provide deeper insights into the move-play-explore nexus and how staff understand and support such activities.

###### Conceptualization of the move-play-explore nexus and the pedagogical principles

The research team collaboratively conceptualized the move-play-explore nexus using the transformational knowledge gained through both deductive and inductive research studies. This effort shaped the pedagogical principles underpinning the move-play-explore pedagogy and will culminate in a conceptual paper that examines the theoretical positioning of the nexus and proposes shared definitions for movement, play, and exploration. We will first establish a theoretical framework for the move-play-explore nexus within the context of ECE pedagogy, public health, and movement sciences, highlighting the importance of transdisciplinarity in bridging these disciplines to foster holistic child development. We will then develop unified definitions for movement, play, and exploration derived from our literature review and empirical findings. The goal is to ensure that these definitions are applicable across different disciplinary perspectives and practical settings in Norwegian kindergartens. By doing so, the project aims to create a shared vocabulary and conceptual understanding that can be used by kindergarten staff and researchers alike in the MoveEarly project.

##### Sample/material

The material included in the scoping review will result from the literature search. The guided tours with post-tour focus groups will be conducted in 3 kindergartens, including 1–2 staff members together with minimum 3 children in 2 different departments (one with younger children and one with older children), summing to 6 guided tours in total. Staff spontaneous filming will be conducted in the same kindergartens.

##### Analysis plan

The scoping literature review approach will be guided by Arksey & O’Malley [111], Pollock et al. [112] and Westphaln et al. [113]. The review will be reported following the Preferred Reporting Items for Systematic reviews and Meta-Analysis extension for Scoping Review (PRISMA-ScR) guidelines [114]. Briefly, we will follow these stages: 1) Specifying the research question, 2) Identifying relevant literature, 3) Selecting studies, 4) Extracting, mapping and charting the data, 5) Summarising, synthesising and reporting the results, 6) Integrating expert consultation through each of the stages. We will use basic qualitative content analysis to identify key characteristics and/or factors related to the concepts movement, play and exploration [115]. Such an analysis is relevant for scoping reviews and in line with MoveEarly´s objective of informing the development of conceptual framework or theory [112].

The data generated from the guided tours and focus groups will be analysed similar to other qualitative data, as described in the process evaluation (see Sect. "Analysis plan" for details). Briefly, the analysis will be inspired by reflexive thematic analysis (RTA) [116] supported by NVivo (QSR Version 12.6) as a data management tool. The purpose of the analysis will be to develop a systematic overview of *themes*—“defined by meaning-unity and conceptual coherence” [116] (p. 77). In this analysis the themes will provide a systematic overview of children and staff’s situated understandings of movement, play and exploration in kindergartens. This analysis will be supplemented by microanalysis [117] to gain a deeper understanding of the move-play-explore nexus and how to develop a child responsive pedagogy facilitating for movement, play and exploration.

#### Co-creation of a continuing professional development program and intervention delivery

##### Background

The objective of the professional development is to enable kindergarten staff to deliver the components of the intervention at the child level (i.e., the move-play-explore nexus). Thus, through theoretical and practical lectures and workshops and sustained support, the professional development aims to increase staff and kindergarten’s competence and motivation to implement the move-play-explore pedagogy. To achieve this, we co-created the professional development with kindergarten staff and other involved stakeholders [70, 71]. The co-creation process followed the working principles suggested by Smith et al. [70] and was placed within an Integrated Knowledge Translation typology, understood as a collaborative process where researchers work with knowledge users (i.e., kindergarten staff) with the aim of increasing the usefulness and impact of the partnership and research. Intervention development was informed by existing research and our research group’s extensive experiences with intervention and education programming. Evidence on implementation strategies of PA interventions is inconsistent and weak [24, 118]. Of particular importance, the development of the MoveEarly intervention was informed by the experiences and process evaluation of *Active Learning Norwegian Preschool(er)s (ACTNOW)* [27], a large-scale cluster RCT where we promoted a multimodal PA intervention for young children through an intensive professional development for kindergarten staff. In ACTNOW, 77 ECE teachers and directors across 23 intervention kindergartens were provided a 7-month professional development delivered face-to-face, supported by online webinars, an online resource, and portable movement and learning equipment. In our experience, a main challenge is to reach *all* staff with ongoing input and support. This calls for a better mix of face-to-face interaction and digital resources to support staff in understanding, implementing, and sustaining the program in their setting. A stronger focus on online resources may provide enhanced reach as it demands fewer resources for follow-up and can be more flexibly tailored to each centre’s context and schedule. Thus, we expand our existing online resource (https://activeinpreschool.com/) by adding an e-learning toolbox to support the professional development program in MoveEarly. Though a 100% web-based delivery could provide superior reach, our experience is that staff find face-to-face delivery of utmost importance. Thus, we decided on optimal solutions of the professional development and resources together with the kindergarten staff and stakeholders.

##### Design and methods

To anchor the project with each kindergarten owner and seek to develop optimal solutions for all, we conducted a co-creation process organized as 3 separate workshops with each of the 3 owners (i.e., the 2 owners involved in the trial and 1 involved in the feasibility study) January-March 2023 (9 workshops in total). Involving the 3 owners and staff with diverse roles and responsibilities (1 director, 1 teacher and 1 assistant from each kindergarten) made it possible to triangulate input from the different groups and from staff with different positions and needs. The main questions that were discussed during these workshops were: 1) How can kindergartens and researchers together develop pedagogical practices promoting movement, play and exploration? and 2) How can researchers support kindergartens in their development work with movement, play and exploration through professional development, digital resources etc.?

The workshops targeted and discussed 3 areas:

- First workshops: Staff needs, wants and expectations. We started with presenting the MoveEarly project, including overall aims and activities of the project. We focused on the aim of the professional development, to upskill staff to better promote movement, play and exploration in their kindergarten on an everyday basis, and the processes and resources needed to support them. Together with the staff, we explored success criteria of the project. The professional development and the resources developed in the ACTNOW project along with our experiences and evaluation was used as a point of departure [119]. However, we facilitated a divergent process on what form the MoveEarly professional development and resources could have, in terms of how it could optimally integrate with kindergartens daily operation and foreseeable organization of their development work. We focused on the staff’s opinion on the value and role of (but not limited to)
  a face-to-face seminars across kindergartens (kindergarten staff visiting the university)
  b face-to-face seminars in kindergartens (researchers visiting kindergartens)
  c collaborative efforts among kindergartens (ways of sharing ideas and experiences)
  d webinars and digital resources (lectures, workshops, toolbox)
  e kindergartens’ written plans (organizational and individual aims and project plans)
- Second workshop: Program feasibility. Based on the input from kindergarten staff in the first workshop, we developed a draft of the MoveEarly professional development and digital resources. The draft was presented to the staff, with the rationale of this model being to verify our understanding of their needs and wants discussed during the first meeting. We discussed pros and cons of the model to facilitate a convergent process towards developing a possible intervention model, including triangulation of perspectives across owners. We focused on how feasible the proposed model was both for kindergartens and researchers. Our understanding of sustainability as an optimal balance of potential effects and spent resources guided this discussion.

Third workshop: Adaptation. Based on the input from kindergarten staff in the second workshop, we refined and presented the final draft of the MoveEarly professional development program and online resources. The focus of the workshop was to discuss how kindergartens could organize their development work integrating with this model; How could kindergartens adapt to this model, or if challenging, how could the model be fine-tuned? Finally, we asked staff to provide input on content and curriculum of the professional development. However, this part has a minor focus because we addressed staff understanding of the move-play-explore nexus and thus competence needs in the conceptual work (see Sect. " Conceptual groundwork for design of the move-play-explore pedagogy (child level)" for details) and because the curriculum would be evaluated and adapted from the pilot study.

The professional development model developed through these seminars was presented to the user advisory board including various stakeholders, who had the opportunity to provide input based on their diverse roles and viewpoints. The final professional development and online resources were shared with staff taking part in the co-creation and kindergarten owners before we started a feasibility study.

##### Sample/material

The sample involved in the co-creation was staff from 7 kindergartens from each of 3 owners, including a total of 63 staff members. Kindergartens was recruited based on diversity in their size and organization.

To describe and evaluate the co-creation process, a minimum of one researcher observed each of the nine workshops and made notes on the topics discussed, the input received from staff, and the process in general. These data were supported by notes created by the staff during the workshops, for example, post-it notes, posters or similar input. Finally, each participant was asked to complete a brief electronic, anonymous survey at the end of the third workshop to provide feedback on how they experienced the co-creation process and what they found valuable and useful.

##### Analysis plan

Quantitative data from questionnaires will be analysed statistically and results described using relevant measures for different types of data. Procedures for analysis of qualitative data will be similar to procedures described for such data in the process evaluation (see 4.3.2 for details). Briefly, the analysis will be inspired by RTA [116] supported by NVivo (QSR Version 12.6) as a data management tool. The purpose of the analysis will be to develop a systematic overview of *themes*—“defined by meaning-unity and conceptual coherence” [116] (p. 77). In this analysis the themes will provide a systematic overview over the themes capturing participants’ perceptions and contributions to the main aspects discussed during co-creation workshops. This means that the initial codes will be anchored in the co-creation main objectives (such as: staff’s needs and expectations, program feasibility and adaptation), while the constructed themes will closely follow the terms and meaning patterns occurring in the empirical material.

A more detailed account of the co-creation process and results will be published in a separate paper.

##### Feasibility study

The professional development developed through the co-creation process (described in Sect. " Intervention and theoretical framework") was piloted with 7 kindergartens over a 7-month period from November 2023 to May 2024. All kindergartens received the intervention, that is, there were no between-group comparisons nor any outcome evaluation of this study. Researchers’ experiences with provision of the professional development and the implementation process in kindergartens along with feedback from staff through focus groups and an evaluation questionnaire was used to fine-tune the intervention before starting the main trial. Main feedback was that kindergartens implemented the intervention well and that staff found participation in the project valuable and were satisfied with the collaboration with researchers. Given the positive feedback from staff, which was largely consistent with our experiences, we did only minor adjustments to the intervention:

- Written work. We provided a stricter template for the project plan. Originally, this task was less concrete to allow for flexibility in structuring and content. However, we learned that this plan needed more structure to ease the writing, to connect more strongly with the pedagogical principles, to target concrete action points and provide milestones of the implementation process to move the project forward with regard to timelines, responsibilities, meeting points etc. This change structured the writing of the plan, but did not limit kindergartens’ flexibility in implementing the intervention.
- Integration of intervention components: We learned that we should put more emphasis on integrating the different intervention components to help kindergartens staff see how they connect. This meant a better integration of online resources and the ideas bank in physical seminars to provide concrete, hands-on experiences with the resource and activities. Thus, during seminars, we provided more information on the online resource (i.e., its intention, content and structure), staff was provided more time to explore it on their own and discuss it with peers, and we included multiple activities in practical sessions so staff could learn how activities worked and how they could be structured and adapted.

### Outcome evaluation

#### Measurements

MoveEarly will include comprehensive measures of child-level effects to capture whole-child development. We will not collect data on adverse events or unintended effects since we do not foresee increased risk of the intervention beyond normal practice. Each data collection will be conducted over a 2-month period and will be organised in close collaboration with kindergartens. All measurements will be performed in the field (i.e., kindergartens) to provide a safe environment for children and avoid travel and disturbance to kindergartens’ practices. Missing data will be minimized by scheduling extra visits to centres if children are not available due to sickness etc.

##### Movement competence and creativity

Movement competence and creativity will be measured using the “River Challenge”, which is being developed for this project. A brief description of its rationale, development and evaluation is provided in Sect. "Analysis plan".

In the River Challenge, children will be invited to take part in three challenges. The first challenge (“Crossing the river”) is based on locomotor and balance skills and the second challenge (“Rescuing the elephants”) on object control skills. In addition, each challenge will capture children’s creativity in and through movements, with a final challenge (“Creating an animal playground”) based on free play to capture creative thinking. The focus of the instruments is on adaptation of these skills to successfully achieve the challenges, rather than measurement of specific skills. Each challenge is presented as a story based on rescuing a variety of animals. This idea is based on Conceptual Playworlds [99], which is a method encouraging children to interact with stories that are personally meaningful to them, rather than responding to adults’ direct instructions. This method encourages adaptability and creativity as there are no specific methods/skills required to be used.

The challenges will be set up within the children’s daily kindergarten setting. The maximum space required will be 2 × 3 m to make it feasible for field testing. The children will be tested one-by-one to capture their own personal skilfulness, adaptability and creativity, rather than being influenced to provide solutions by others. Thus, tests will be conducted in a separate room or space. Tasks will be completed in a standardised order, using approximately 20 min per child. The test teams will consist of one instructor who tells the story, while a separate assessor observes and scores the child’s performance. Both challenges have eight levels (i.e., items), which will be scored based on successful or unsuccessful achievement. There will be no specific method/skills required to achieve each challenge, however detailed and standardized descriptions of what is ‘successful’ and ‘unsuccessful’ will be a fundamental aspect of the scorers' training. Scores are summed for each item and challenge.

Challenge one will be set up as a river crossing where children are invited to cross from one riverbank (a set of folded mats) to another, using stepping stones to rescue a variety of objects. The story suggests that some frogs have gone on an adventure across the river and need to be rescued. The children then use the stepping stones to cross the river, without falling in, pick up the differing sizes of objects and bring them back across. At each level, the number or stability of the stepping stones is reduced and/or the objects are changed (from picking up 2 small bean bags to a yoga ball and a heavy medicine ball). Children need to demonstrate a variety of different locomotor and balance skills and adaptable solutions to successfully manage the challenge as the environment changes.

Challenge two will be set up as a situation where children are invited to retrieve and throw different sized balls at different sized objects and to use different equipment to retrieve moving objects. The story used to engage children is one of various elephants (represented by various sizes of yoga balls) have become stuck in the mud and need help to get out. The suggestion is to throw a variety of sizes of balls to the target to move the yoga ball. The size of each ball to be caught and thrown and the size of the target is reduced to increase difficulty as each level progresses. In the conclusion of this challenge the children are asked if they want to catch some fish swimming down the river. These are small balls rolled down a ramp, to standardize the speed, and the children try to scoop the balls up in a short fishing net first, followed by a longer fishing net, from the riverbank. Children need to demonstrate a variety of different object control skills to successfully manage the challenge as the environment changes.

Movement creativity challenges are also included where children are encouraged (through the story) to find different solutions to movement challenges. The creative thinking challenge involve free use of a set of predefined resources (mats, hoops, sticks, cones, ropes, paper cups, blankets and cloths pegs). This challenge will not include any instructions besides an idea to inspire creativity. The child will have up to 5 min to create a play environment/playground for the animals introduced in the first challenges. Creativity will be scored based on fluency and originality. Fluency refers to the number of different solutions the child finds to achieve the task. Originality refers to the type of solution the child demonstrates on a scale from no response to predictable, less predictable, new, unique or original response. These categories will be determined using the data from the baseline testing. The lower the frequency of a response, the higher the originality score will be [120].

More details about the test, scoring procedures and measurement properties will be published in a separate paper.

##### Physical activity, sedentary time and screen time

We will assess PA using the ActiGraph GT3X + accelerometer [121], which is the most used and validated accelerometer in the field [122, 123]. We will instruct children to wear the accelerometer on the right hip 24 h per day for seven consecutive days, except during water activities like swimming and showering. Accelerometers will be initialized using a sampling rate of 30 Hz. Files will be analysed using a custom-made script in MATLAB applying 1 s epochs to correctly capture the lower and higher intensity levels [124, 125]. Consecutive periods of ≥ 20 min of zero counts will be removed as non-wear time [126, 127]. We will report results for overall PA level (counts per minute (cpm)) and apply the previously established and validated Evenson et al. cut points [128, 129] to determine minutes per day spent sedentary (SED) (< 100 cpm), in light PA (LPA) (100–2295 cpm), in moderate PA (MPA) (2296–4011 cpm), in vigorous PA (VPA) (≥ 4012 cpm), and in moderate-to-vigorous PA (MVPA) (≥ 2296 cpm). As appropriate, sensitivity analyses will be conducted using the Pate et al. cut points [130].

Adding to the traditional PA intensity profile, we will include a higher resolution PA descriptor of time per day spent in 17 narrow intensity intervals across the intensity spectrum; 0–99, 100–999, 1000–1999, …, 14,000–14,999, and ≥ 15,000 cpm. Similar to previous applications [131–134], this dataset will be used to investigate more nuanced PA signatures associated with relevant outcomes.

To assess intervention effects on overall PA levels, as well as in care and out of care to assess possible compensatory behaviour outside the kindergarten domain [20, 135], we will assess PA levels over the full week, weekdays and weekend days separately, as well as PA for the whole day (06:00–21:59) and for care hours (08:30–15:30) and afternoon hours (15:30–21:59) on weekdays. Wear-time requirements will be ≥ 8 h/day and ≥ 3 weekdays + ≥ 1 weekend day for whole days and ≥ 5 and ≥ 3 h/day of monitoring for ≥ 3 weekdays for care hours and afternoons, respectively.

We will assess screen time using a parent questionnaire adapted from the Sunrise study [136].

##### Physical fitness

The fitness assessments include two items (handgrip strength and standing long jump) from the Assessing FITness in PREschoolers (PREFIT) test battery [137] and one item (Supine Timed Up and Go) from the SUNRISE study [136]. The PREFIT tests, adapted from the Assessing Levels of Physical Activity field-based fitness tests for the assessment of health-related physical fitness in children and adolescents, have shown good reliability in young children [138–140]. A hand dynamometer with adjustable grip will be used to measure hand grip strength (TKK 5001 Grip A, analogue model [141], measurement range 0–100; Takey, Tokio Japan), applying a grip‐span of 4.0 cm [142]. The mean of the best of two trials for each hand will be used. Standing long jump, instructing children to jump as far as possible with both feet together, will be used to measure lower body explosive strength. The best of two trials will be used. Speed-agility will be assessed using the Supine Timed Up and Go test, where children is instructed to lie on their back with their feet on a line, get up as quickly as possible on ‘Go’, run 3 m, to a wall and touch it and return to cross the start line. The best of two trials will be used.

##### Anthropometry and demography

Body weight to the nearest 0.1 kg will be measured using an electronic scale (Seca 899, SECA GmbH, Hamburg, Germany). Height to the nearest 0.1 cm will be measured with a portable stadiometer (Seca 217, SECA GmbH, Hamburg, Germany). Body mass index (BMI, kg/m^2^) will be calculated based on the criteria proposed by Cole et al. [143] and children classified as normal weight (including underweight), overweight, or obese. Demography will be measured by parent questionnaires and includes socioeconomic status (parents’ education and income), children’s, parents’, and grand parents’ origin, children’s birth weight, parents’ weight status and parents’ PA.

##### Sleep

We will assess children’s sleep using 24-h accelerometry and a parent questionnaire. From accelerometry, we will estimate sleep using an algorithm developed and validated to identify sleep/bedrest from waist-worn triaxial accelerometers in 3–6-year-old children [144]. First, the *PhysicalActivity* (v0.2.4) R package is used to remove non-wear periods, defined as ≥ 90 min of consecutive zero counts allowing 2 min of interruptions. Second, the *PhysActBedRest* (v1.1) R package is used to determine sleep using vector magnitude data at 60 s epochs [144]. This algorithm has good sensitivity (0.94), specificity (0.97), and accuracy (0.95) as compared with visual identification of accelerometry-measured sleep periods. In the questionnaire, used in the Sunrise study [136], parents report children’s sleep duration including nap time, when they go to bed and when they wake up, and the consistency of these patterns. Children’s sleep quality is rated on a 1–7 scale.

##### Social-emotional health and self-regulation

To assess social-emotional health, teachers will complete the one-sided Strengths & Difficulties Questionnaire (SDQ, 4–17 years) for each child to provide a brief measure of children’s psychosocial strengths and problems. The SDQ asks about 25 attributes (10 positive, 14 negative, and 1 neutral) and includes five subscales (five items each): *1. Emotional Symptoms Scale; 2. Conduct Problems Scale; 3. Hyperactivity Scale; 4. Peer Problems Scale;* and *5. Prosocial Scale*. Scales 1–4 can be summed to provide a total difficulties score. SDQ uses a 3-point Likert scale (“not true”, “somewhat true” or “certainly true”) to indicate to which extent each attribute applies to each child [145, 146]. The construct validity of the teacher version of SDQ for young children has been demonstrated in several studies [147–150] and has been shown to have better internal consistency and test–retest reliability than the parent version for kindergarten and school children [147, 148].

Self-regulation will be assessed by a structured observation of a child’s performance on the Head-Toes-Knees-Shoulders Revised (HTKS-R) task [151], which includes a downward extension of the original HTKS [152]. The test assesses to which extent a child can use and integrate executive functions to control actions, pay attention and remember instructions [153–156]. To respond correctly, children must listen to instructions, remember and execute gross motor movements according to directions, and inhibit pre-potent incorrect responses. In the first part (10-items), the command “touch your head” requires the children to touch their toes and vice versa, while in the second part (10-items) the command “touch your shoulders” requires the children to touch their knees and vice versa [153]. If a child reach a minimum of 4 points on both levels (2 points are given for a correct response, and 1 point for a self-corrected response), they will be given a third part (10-items), where the paired rules are switched (i.e., head pairs with knees and shoulders pairs with toes) [152]. The HTKS-R also includes a Part 0, where children are asked to say (instead of doing) the opposite of what is instructed [151]. Predefined instructions will be given verbally, without feedback on responses. To familiarize with the test several practice trials are provided at each level [155]. The HTKS is easy to administer, requires few materials and has shown construct validity in European samples [154]. Self-regulation will be scored as the sum of correct (2 points) and self-corrected (1 point) responses over the four parts (range 0–118 points). The project group translated the HTKS-R form to Norwegian.

##### Early academic performance

We will use “*The Expressive Vocabulary”* for language development [157] and *“The Numbers task”* for early math skills [158], both iPad tasks from the Early Years Toolbox, as proxies for early academic performance. Both tasks have shown good convergent validity and reliability. The Expressive Vocabulary task includes 55 items where children are asked to verbally produce a correct label for depicted nouns and verbs. The administrator records the responses (correct/not correct) in an iPad app. In cases of an incorrect response, the administrator will ask children “what else might this be called” until a correct response is given or that the child is unable to produce the target word is indicated. The test will stop if children provide six consecutive incorrect responses. Expressive vocabulary will be scored as overall accuracy [157].

The Numbers task includes 79 items covering numerical concepts (e.g., many), spatial and measurement concepts (e.g., tallest), counting subset (e.g., counting dogs interspersed with cats), matching digits and quantities (e.g., understanding the correspondence between: spoken number *two*, the digit *2*, and its corresponding quantity), number order (e.g., identifying the missing number in a number line), ordinality (e.g., identifying higher/lower numbers), cardinality (e.g., identifying what is 1 st, 2nd, etc.), subitizing (e.g., a rapid appraisal of quantities as having more or less), patterning (e.g., identifying and completing a pattern), and numerical word problems and equations. Children respond by tapping the iPad screen or verbally answering questions in the app. The app includes automated start rules based on the age of the child. The test will stop if children provide five consecutive incorrect responses. Early math skills will be scored as overall accuracy [157].

##### Playfulness, explorative behaviour and well-being

Each of the constructs playfulness, explorative behaviour and well-being will be measured by 7 items using the “Children’s Involvement and Well-being in Movement, Play and Exploration Questionnaire”, developed in this project. A brief description of its rationale, development and evaluation is provided in Sect. "Development of measures of movement competence, creativity, playfulness, explorative behaviour and well-being in young children.". Kindergarten staff will be asked to score the child’s observed behaviour within the past month to capture how children generally behave and will avoid any specific events which may have an impact on the child. The child will be scored for each statement on a 5-point Likert Scale from “never” to “almost always”. Scores are summed for each construct or scored alternatively, given the instrument's construct validity. More details about the instrument, scoring procedures and measurement properties will be published later.

#### Analysis plan

##### Primary aim

To test the study’s effectiveness, the main analyses will employ an intention-to-treat principle, including all participants and kindergartens originally allocated to their respective groups [159, 160]. To study efficacy, secondary analyses will be limited to kindergartens that exhibit acceptable intervention adherence. We will seek to minimise missing data [161]. To allow for investigation of missing data mechanisms and thus assumptions underlying the statistical analyses, we will provide a detailed account of reasons for missing observations using suitable flow charts [81]. We will include attrition analyses to investigate whether missing data are related to child characteristics. In the analyses, missing data will be handled using linear mixed models or structural equation modelling (SEM) [162–165].

We will determine intervention effects by testing for time*group interactions adjusted for baseline levels of the outcome [166]. We will account for the cluster effect of participant and kindergarten using linear mixed models [167] (or generalized linear mixed models, dependent on distributions) or multilevel SEM for mediation analyses [164, 168]. The primary analysis will not be adjusted for covariates (beyond baseline levels of the outcome), while the per-protocol analysis will be adjusted for differences between groups, as appropriate.

Interpretation of study effects will be based on p-values (i.e., a p-value ≤ 0.05 is considered statistically significant), effect sizes and patterns of effects across variables; the latter will be considered of greatest importance when drawing study conclusions. Consistent with this approach, p-values will not be adjusted for multiple testing. Main analyses will be performed using IBM SPSS v. 30 or later versions (IBM SPSS Statistics for Windows, Armonk, New York: IBM Corp., USA) and MPLUS v. 8 or later versions (Muthén and Muthén, Los Angeles, USA). Reporting will be guided by the CONSORT statement [81, 169].

###### Main and secondary outcomes

Given that the broad MoveEarly intervention is targeted at promoting whole-child development, designating one primary outcome a priori is challenging. As a single test is not capable of capturing multiple aspects of a child’s development, we will determine effects across multiple relevant outcomes. Whilst regarding children’s movement competence and creativity as priority outcomes, we are cautious about defining the River Challenge as the main outcome given its unknown measurement properties and lack of data to allow a sample size calculation. If the River Challenge demonstrates acceptable measurement properties, we will designate movement competence and creativity as the main outcomes, and other aspects of children's development as secondary outcomes.

###### Secondary aims

####### **Secondary analyses of intervention effects**

Evidence shows that PA interventions in school may be most beneficial for those most in need [170, 171] and more beneficial for boys than for girls [172]. Thus, we will include analyses of effect moderation by sex, age, socioeconomic status, and baseline levels of outcomes. We will also test for mediation effects to explore possible mechanisms, given significant intervention effects. Mediation analyses may include half-longitudinal (2 timepoints) or full (3 timepoints; given available data for relevant mediators) mediation models using SEM [164].

####### **Association analyses**

Cross-sectional and longitudinal association analyses will be used to study correlates and determinants of child behaviours and development. Longitudinal analyses will include both groups (intervention and control) to increase power, if there are trivial intervention effects. Growth models will be used to determine reciprocal relationships among variables over time [164]. In addition to standard regression models, we will use multivariate pattern analysis to explore multivariate association patterns among the included variables [133, 173]. Multivariate pattern analyses will be performed using the package *mvpaShiny* v. 0.0.0.9000R [173] in R v. 4.3.2, [174]/RStudio v. 2023.09.1 [175], while other analyses will apply the same statistical models and softwares as specified above.

#### Development of measures of movement competence, creativity, playfulness, explorative behaviour and well-being in young children

##### Background

To focus on young children’s holistic development rather than school readiness, new measurement tools are required which evaluate relevant aspects of children’s movement, play and exploration. We will develop measures that capture young children’s skillfulness, adaptability and creativity in and through movement, along with their playfulness, explorative behaviour and well-being. The new or adapted instruments will focus on how children adapt and explore movement solutions in a range of contexts or scenarios and how kindergarten staff consider their behaviours in care, therefore making these tools, not only valid and reliable, but also feasible and relevant.

Whilst there are several validated instruments that assess fundamental movement skills [176], their scale of analysis is the organism with a focus on only one optimal movement pattern in a closed decontextualized environment. Thus, the nature and structure of these measures remove the embodied and embedded nature of play [49, 75]. New measures are needed that assess how young children can adapt and extend movement solutions in a range of movement, play, and exploration contexts. To be specific and relevant to kindergarten-aged children, this requires measures that are more representative of play. We have developed a measurement instrument based on the theoretical concepts of ecological dynamics, where environmental and task constraints encourage the emergence of adaptive behaviours [177], and also on the theory of Compound Flexibility [178], which links adaptability in a child’s play environment with opportunities to explore and the effect these can have on children’s well-being. The instrument therefore includes aspects of movement adaptability encouraged by changes to the environment and creativity encouraged by substantial flexibility in the child’s responses.

There is limited research into the measurement of play and exploration. This could be because the concepts of play and exploration are difficult to define and measure [179]. However, the outcomes of such behaviours could be measured. For example, during play, aspects such as playfulness, enjoyment and engagement [180, 181] could be observed. Capturing a child’s well-being can be challenging as there are broad and differing definitions of this concept, with many including multiple domains of well-being [182]. We developed an instrument to capture children’s well-being within the context of movement, play and exploration in kindergartens. This would be most suitably captured within a child’s natural environment, rather than in a standardized laboratory situation. Thus, we developed a staff-report observation instrument which aims to capture kindergarten staff’s perceptions of children’s playfulness, exploratory behaviour and well-being.

##### Design and methods

A systematic literature review was undertaken to learn what has already been done with regard to the measurement of movement, play and exploration. This was registered with PROSPERO (reference number CRD 42023396648). The analysis and reporting will be based upon the Preferred Reporting Items for Systematic Reviews and Meta-Analyses (PRISMA) statement [183]. We searched for publications which investigate the validity and/or reliability of any measurement instruments assessing play, exploration and/or creativity and instruments assessing movement including play, exploration and/or creativity.

The laboratory assessment instrument (River Challenge) was guided by and extends tools such as the Movement Ability Test [184] and the Thinking Creatively in Action and Movement test [185]. We planned that the new instrument should have the following features:

- The design of several carefully constructed playful challenges will be inspired by observations of children’s free play, ensuring these are relevant to the age group involved. The challenges will be based on stories to help engage children in the challenges and to encourage playfulness.
- Through these challenges, each of which has key information and constraints for children to perceive and regulate action, the play environment will invite children to functionally apply and adapt various locomotor, object control, and balance skills.
- Scoring of movement competence will be based on functionality (success or not, irrespective of how the challenges are solved), which is a key concept of ecological dynamics. Scoring of creativity will be based on fluency and originality.

The staff-report observation instrument (Children’s Involvement and Well-being in Movement, Play and Exploration Questionnaire) was designed to capture facets of children’s playfulness, exploration, and well-being in everyday play settings. Existing measurement scales exist, such as The Children’s Playfulness Scale [180], which aim to capture playfulness, and the Leuven Involvement and Wellbeing Scale [186], which aims to capture well-being. However, as the intention of the research is to capture exploration, playfulness and well-being, a revised staff report observation instrument is justified. The staff-report observation instrument was designed as a multi-item questionnaire for staff to reflect on the children’s involvement, experiences and reactions within the kindergarten environment over the past month, where each child’s behaviours are scored on a 5-point Likert scale.

The development of both instruments builds on our research team’s extensive experience with test batteries that have been used in large studies and historical and cultural analyses of children’s play and exploration activities. The two instruments was developed using the process for instrument development suggested by de Vet et al. [187]. Each instrument was piloted, revised and adapted before field testing. We will determine measurement properties as recommended by the Consensus-based standards for the selection of health measurement instruments group [188], using in-person and video scoring of children.

##### Sample/material

The material included in the systematic review will result from the literature search. Observations of children’s free play and development and testing of initial instrument ideas was based on small convenience samples of children. For evaluation of measurement properties of the laboratory assessment instrument and the staff-report instrument, we included 139 children aged 4–5 years in measurements on two occasions separated by 1–2 months and the kindergarten staff who work with them completed the questionnaires on these two occasions.

##### Analysis plan

For the systematic review, the studies identified will be screened and selected in line with the inclusion and exclusion criteria by two independent authors. Initially a title search will be undertaken, followed by abstract screening and full text screening. A valid reason will be given for excluded studies. The method of analysis will be a narrative review to capture the relevant information regarding validity and/or reliability of existing instruments. For River Challenge, we will determine test–retest, inter- and intratester reliability, as well as structural validity. Analyses of reliability will be performed in SPSS. Test–retest reliability will be assessed over 1–2 months using Bland–Altman plots showing potential learning effects (bias) and random variation (95% Limits of Agreement) [189] and ICCs derived from a two-way mixed model using a consistency definition [187, 190]. Inter- and intra-tester reliability will be based on video-scoring of 20 children, representative of the larger sample with respect to age, sex and performance. Assessors will rate the same videos on two occasions, from which within-subjects or between-subjects ICCs will be derived from two-way mixed models using a consistency definition (excluding variation for assessors and time, respectively) [187, 190]. Structural validity will be determined using Confirmatory Factor Analysis (CFA) [191] in Mplus. Using CFA, we will compare the data to the hypothesized measurement model and evaluate model fit using suggested model fit indices [192]. If the model fit is not acceptable, modification indices with theoretical justifications will be used to adjust the model. If the model is still not acceptable, Explorative Factor Analysis (EFA) will be used to explore alternative models. We will test for invariance across sex in the accepted model [191].

For the Children’s Involvement and Well-being in Movement, Play and Exploration Questionnaire, we will determine internal consistency, intra-tester reliability and structural validity. Internal consistency will be determined using McDonald’s Omega [193] on the separate constructs after validating the structure of the instrument. Intra-tester reliability will be determined using ICCs derived from a one-way mixed model using an absolute agreement definition [187, 190], based on scores on two occasions from the staff member who knows the children in a given department best. Given the design, it is not possible to separate measurement errors over time for children’s behaviours (test–retest reliability) and staff’s judgements (intra-tester reliability). Yet, given that there is no intervention in between of the two measurement timepoints, we regard significant changes in children’s behaviours over this short period less likely. Inter-tester reliability will not be assessed, as there are few staff members who know the children well enough to do this assessment. Structural validity will be determined using CFA as detailed above.

### Process evaluation

Given that MoveEarly is framed within a “realist RCT” approach [73], a complex systems perspective will be used to explore the deeper (sociological) mechanisms by which the kindergartens interact with the intervention [68]. The initial program theory, described in Sect. " Intervention and theoretical framework", states that by enhancing staff competence and motivation to integrate the move-play-explore pedagogy into kindergarten’s daily operations, we expect to find positive effects on children’s health, development, well-being, and learning. This causal pathway, and how it varies according to individual and contextual factors, will be examined in the process evaluation, which seeks to provide a general overview as well as a deeper insight into how staff and children in the intervention kindergartens respond to the MoveEarly professional development and integrate the move-play-explore pedagogy into staffs’ and children’s daily practices. Thus, the process evaluation will explore what works for whom, under what circumstances, and why, within the MoveEarly intervention. As suggested by McGill et al. [194], we will use a 2-phase framework for the process evaluation that seek to assess change over time from a complex systems perspective. The first phase will involve producing a description of the system and identifying hypotheses about how the system change in response to the MoveEarly intervention. The second phase will involve adapting the planned evaluation approach in accordance with the pathways of the emergent findings.

#### Samples and methods

**Fig. 5:**
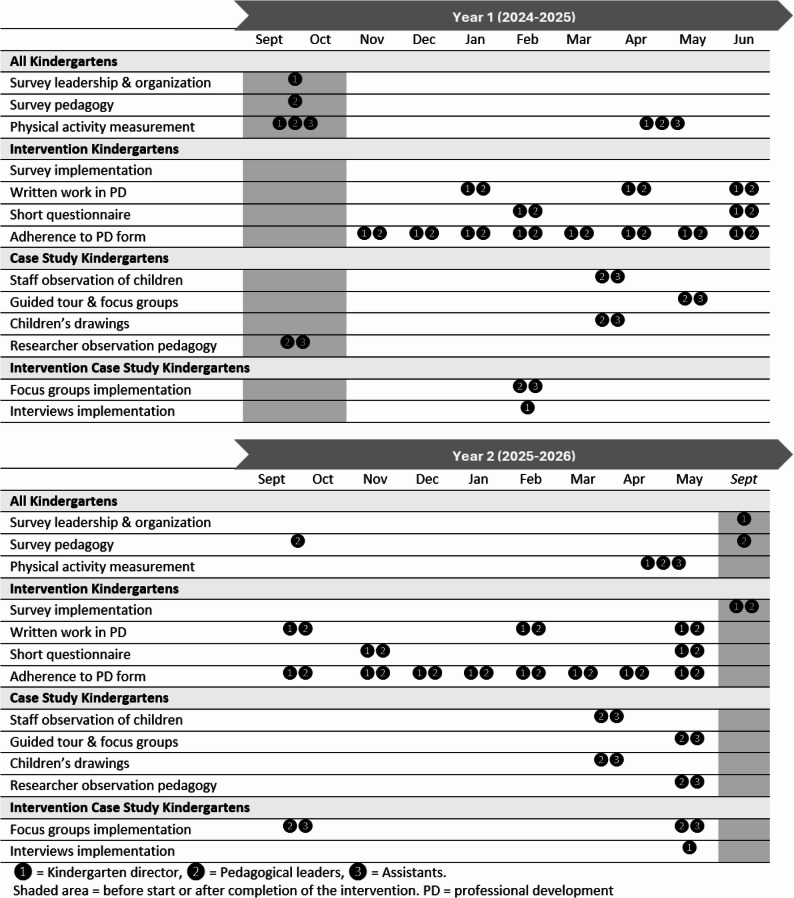
The timeline of the data collection in the process evaluation

The process evaluation will draw on multiple methods to triangulate data and user perspectives [78] and includes four samples. The first sample includes all kindergartens participating in the study (i.e., both intervention and control), the second sample includes all intervention kindergartens, the third sample includes selected kindergartens invited to take part in a multi-site case study (i.e., both intervention and control), and the fourth sample includes only the intervention kindergartens taking part in the multi-site case study. While the research including all kindergartens will test between-group effects and describe implementation measures in the MoveEarly intervention, the multi-site case study will use an ethnographic approach to provide an in-depth understanding of how the intervention evolves and builds momentum in kindergartens [195]. An overview of the methods and timelines for the data collection in the process evaluation is provided in Fig. 5.

##### Sample 1: All kindergartens

Data collection in sample 1 includes a questionnaire about the kindergarten’s leadership and organization, a questionnaire about the kindergarten’s pedagogical practices and measurement of staff PA levels.

Kindergarten’s leadership and organization will be assessed by a questionnaire completed by directors at baseline and at 24-month follow-up. Focus areas are staff’s perceptions of the national policy, their local context and the kindergarten’s daily organization. The baseline questionnaire will be used to sample kindergartens to the multi-site case study.

Self-assessment of pedagogical practices will be performed by pedagogical leaders of each department using a brief custom-made questionnaire, guided by the pedagogical principles of the intervention (Sect. "Child level – A move-play-explore pedagogy") and a custom-made environment rating scale developed to assess pedagogical practices by researcher observation (Sect. "Sample 3: Multi-site case study"). The questionnaire has 25 items assessing the kindergarten’s indoor physical environment (items 1–6), the kindergarten’s outdoor physical environment (items 7–12) and the kindergarten’s pedagogy (items 13–25) and will be completed at baseline, 12- and 24-month follow-up.

Staff’s PA level will be assessed using accelerometry, performed simultaneously with the three child measurements at baseline, 7- and 18-month follow-up. We will use the same procedures as detailed for children, except using another criterion for non-wear time (≥ 60 min of zero counts) and using the commonly applied adult Troiano cut points to determine intensity-specific PA (SED < 100 cpm, LPA 100–2019 cpm, MPA 2020–5998 cpm, VPA ≥ 5999 cpm, and MVPA ≥ 2020 cpm) [196]. Data will primarily be analysed using short epochs (1 s) to allow for capturing short intermittent bursts of PA consistent with children’s activity patterns, but we will also use 60-s epochs for comparison with previous adults studies [197, 198].

##### Sample 2: Intervention kindergartens

Data collection in sample 2 includes written work by staff submitted as part of the professional development, registration of adherence to the professional development, multiple short implementation questionnaires and a project evaluation questionnaire. Contact points between researchers and kindergartens will be logged.

Several types of written work (project plans, reflection papers and project reports) will be collected from the kindergarten internal project group to assess the visibility and formalisation of the MoveEarly intervention in the intervention kindergartens. Project plans will include each kindergartens’ own aims for the development work, the planned approach to integrate the MoveEarly pedagogical principles into their daily practice (e.g., whether principles and/or activities will expand, extend and/or enhance children’s opportunities [84]) and a description of the organization of the process, including milestones, leadership and responsibilities. Reflection papers will include a discussion about children’s and staff’s experiences with the move-play-explore pedagogy in relation to the pedagogical principles based on plans, videos, pictures and/or observations. Project reports will include staff’s reflections regarding their experiences of successes and challenges during implementation of planned aims, pedagogical approaches and institutional organization, with the overarching aim of developing action points for improved future practices.

Adherence to the professional development will be registered throughout the intervention period. These data will include attendance to all physical and digital meetings and submitted written work. Additionally, researchers will score each kindergarten on a 5-point Likert scale in terms of the 1) director’s and project group’s leadership and 2) staff engagement. Primary focus areas will be level of concretization of tasks, plans and milestones in written work and meetings, and involvement of all staff members in implementing the intervention.

The kindergarten project group will complete multiple short questionnaires to rate their experience of collaboration with researchers, resources provided them, and implementation of the intervention in their kindergarten, at all physical seminars except for the kick-off. Questions will be tailored on each occasion according to formal and informal feedback from kindergartens during the project period.

At 24-month follow up, the kindergarten internal project group will be asked to complete an evaluation questionnaire. The following three domains will be examined: 1) Implementation, 2) mechanisms of impact and context and 3) sustainability of the new practice in the kindergartens’ daily operations [199]. Specifically, we will assess how staff experienced participation in the intervention, including their experience with the move-play-explore pedagogy, satisfaction with the professional development, digital resources, and collaboration with researchers, as well as barriers and facilitators of their kindergarten’s development work. The questionnaire will be informed by researchers’ experiences with running the professional development and preliminary findings from the process evaluation.

##### Sample 3: Multi-site case study

We will purposively sample 8 intervention kindergartens and 4 control kindergartens to participate in the multi-site case study. Kindergartens will be sampled for diversity in terms of the extent to which the kindergarten seems responsive to the intervention (assessed by baseline questionnaire and initial project description), urban and suburban locations, outside and inside facilities, and the kindergarten’s owner and profile. This approach allows a rich inquiry into intervention implementation in several different, naturalistic settings, and thus allow a more in-depth understanding of the phenomena than a single case can provide [200].

As the intervention relates both to the kindergarten staff and children, both groups will be included in the qualitative studies. In this way, we seek to better understand children’s whole bodily repertoires, and the conditions for them, when children move, play, or explore their environments. Considering children’s tendency in being enactive and easily engaging in activities, showing a genuine interest and enthusiasm (i.e., agency), we will study children’s behaviour as meaningful projects that extend out into the world and are deeply embedded with the environment. We will intertwine in our design both *child perspectives* and *children’s perspectives* as defined by Sommer, Pramling Samuelsson and Hundeide [201]. We understand *child perspectives* as “created by adults who are seeking, deliberately and as realistically as possible, to reconstruct children’s perspectives, for example through scientific concepts concerning children’s understanding of their world and their actions in it” [201] (p. 22). However, although “child perspectives attempt to get as close as possible to children’s experiential world they will always represent adults’ objectification of children” [201] (p. 22). The *children’s perspectives* on the contrary “refer to the perceptions of the non-adult subjects themselves” (p. 22), and capture children’s ‘here and now’ experiences, perceptions, and understandings of their physical and social environments.

A range of methods will be used to capture kindergarten practices, including staff, children’s and child perspectives:

 Staff observation of children

Observation is a significant part of kindergartens’ pedagogical practices [202–204] and may have a summative [204] or more dialogical [203] character in different ECE traditions. While the summative approach focuses children’s progress in development, the dialogical approach highlights a process of documentation that stimulates teachers' reflection over own practice [203] by trying to understand it from the perspective of the child/children [203, 205, 206]. The dialogical perspective rejects the accountability approach, while highlighting the importance of teachers’ subjectivity, reflection and meaning-making in attempting to widen and understanding children’s (re)actions and their own professional practice. The dialogical perspective is in line with the Nordic approach to ECE [202].

In Norway, the established methods for observation in ECE are a) continuous notes—*løpende protokoll*, b) story from practice—*praksisfortelling*, and c) participatory observation—*deltagende observasjon* [207]. What differs in these methods is the distinction between empirical notes and interpretation. While for *continuous notes* and *participatory observation* it is important to make notes as objectively as possible (and invite diverse interpretations in the next stage), *story from practice* allows for immediate subjective interpretations (e.g., while a teacher in continues notes would note a child is smiling, a story from practice would note a child being happy). In line with the technological development, the ECE sector has also made use of video-based observation, which allows for a more objective understanding of their own practices both from professional, ethical and child perspectives [208]. This approach turns out to be particularly beneficial in evaluation of complex interactions [209] and for self-evaluation [210] and self-reflection [211].

While we recommend video-observations, continuous notes or participatory observation, teachers will be allowed to choose among different observation methods to maximize chances of gathering observational data. Regardless the chosen observation method(s), teachers will be asked to:

- Choose 2 children with different needs; (*a*) a child who easily engages in activities and *b*) a child who often needs extra support to participate in activities.
- Conduct observations on the children’s experiences of the move-play-explore pedagogy 3 times a week over a period of 2 months towards the end of the first and second years of intervention
- Share their interpretations of the children’s experience during the interview following the guided tours.

«Guided Tour»—video-based observation of situated practices

Guided tour is a participatory research technique developed for studying embodied and situated knowledge/experience [108, 109]. The participant(s) are positioned in the roles of experts who guide the researcher around a space that is familiar and professionally or personally meaningful for them. The researcher is positioned as a visitor totally dependent on the participant(s)’ knowledge to move around the visited space. Such positioning gives the researchers possibility to access deeply situated and embodied knowledge, mediated through the stories, actions and body language of the research participants. Video and audio recording of such visits allows to document the verbal and nonverbal storytelling on the “local ecologies” [110], revealing the way the participants interact and experience the space, and what meanings they attach to different areas.

In MoveEarly, the guided tours will be implemented to access the embodied, situated and practiced understandings of the project’s main concepts: *movement, play, and exploration*. In each kindergarten, 1–2 of the kindergarten staff together with minimum 3 children in 2 different departments will guide the researchers around and show, tell, and demonstrate how and where they move, play, and explore. Studying the verbal-nonverbal interactions among participants and the surroundings, will allow researchers to reflect on the embodied, situated, lived, and practiced understandings of the concepts move, play, and explore and thus support development of the pedagogical design. The guided tours will be informed by the guided tours performed in the conceptual groundwork for pedagogical design (4.1.1.2) and other parts of the process evaluation where change of the kindergartens’ conceptualisations of move-play-explore will be investigated. After the tour, focus groups with kindergarten staff will allow us to study staff’s reflection over the places/spaces, activities and understanding that appear and become visual/verbal during the guided tour.

 Children’s drawings

As the children are important actors in the kindergarten context, we need to develop ways of capturing their experiences. Being aware that the verbal code is only one, and often not the optimal, way for children to express their experiences and meanings, we will employ drawing as a method that allows another form of expression [212, 213]. Since drawing is one of the kindergartens’ daily activities, it will not take children away from their daily life in the kindergarten into, for example, an artificial interview setting. It will capture children’s experience of the kindergarten activities, through a salient, familiar activity. Once a week over two months in the end of the first and second intervention years, preschool children (i.e., last year of the kindergarten) will be asked to draw “the most joyful activity that they have participated in during this week”. The drawings will create a base of visual data documenting children’s perception of their best activities, including moments of move-play-explore as (hopefully) implemented in the daily kindergarten practice.

 Observation of the movement environment

We will use a custom-made observation instrument specifically capturing the move-play-explore pedagogy to quantitatively assess staff’s pedagogical practices. This instrument will be based on the pedagogical principles underpinning the child level intervention (Sect. " Child level – A move-play-explore pedagogy") and will focus on observable indicators, as information received from staff logs or interviews is difficult to obtain and may introduce errors. The instrument includes 8 items and assesses 2 dimensions of the environment: Physical enrichment (items 1–4) and Child-staff-interactions (items 5–8). Each item is scored from 1 to 7 based on multiple indicators, equivalent to the Movement Environment Rating Scale [214] and other similar environment rating scales. Observations will be performed by trained researchers for a full day (08:30–15:30) within each kindergarten department. More details about the instrument will be published in a separate paper.

##### Sample 4: Intervention kindergartens in multi-site case study

Data collection in sample 4 includes focus groups and structured interviews of participants in the internal project group.

Focus groups will be used to collect data on teachers’ and assistants’ views of the fidelity, reach and acceptability of the professional development curriculum and the move-play-explore pedagogy, contextual barriers and/or facilitators of implementation; staff’s previous experience from participation in research and professional development programs, views on the fit between the aim of MoveEarly compared to local and national kindergarten policies as well as the social dynamics among staff and children. The focus groups will also address how the director and project group has led professional learning community’s participation in the intervention. Interviews will be conducted physically at 3-, 10- and 18-month follow-ups.

Structured interviews will be used to collect data on the internal project group’s views of the fidelity, reach and acceptability of the professional development curriculum and the move-play-explore pedagogy, contextual barriers and/or facilitators of delivery, staff’s previous experience from participation in research and professional development programs, views on the fit between the move-play-explore pedagogy and local and national ECE policies as well as the social dynamics among staff and children. Interviews will be conducted digitally with directors at 3- and 18-month follow-ups.

#### Analysis plan

##### Observations of children and situated practices, interviews and written work

The analysis of data from observations of children and situated practices, interviews and written work submitted by the teachers during the professional development will be inspired by RTA [116] and supported by NVivo (QSR Version 12.6) as a data management tool. Prior to the analyses, interviews and video data (cut into shorter fragments of 10–15 s [110]) will be transcribed such that all qualitative data has a textual form.

In line with the RTA approach [116], the ultimate analytical purpose of the analysis will be to develop a systematic overview of *themes*—“defined by meaning-unity and conceptual coherence” (p. 77). In this analysis the themes will provide a systematic overview over the themes capturing children’s and staff’s actions, experiences, and meanings related to the move-play-explore pedagogy and professional development. This means that the initial codes in the analytical work will be anchored in the trial research questions, the intervention program theory, key findings from the empirical data from the ACTNOW trial, insights from the pilot study and pedagogical content of the intervention (movement-play-exploration). Further, themes will be developed inductively to capture nuances within codes or relevant phenomena that is not connected to a priori code. In the process of searching and reviewing the themes, reflexivity will be highly important. By reflexivity in thematic analysis, Clarke and Braun [116] mean a critical reflection over how disciplinary, theoretical and personal assumptions shape the analytical choices, that as a result delimit knowledge that is produced. As MoveEarly is an interdisciplinary project engaging researchers from different disciplines, research paradigms, and traditions, reflection and transparency regarding the criteria for the final chosen themes of analysis are important not only for the analysis but also for research ethics.

**Fig. 6:**
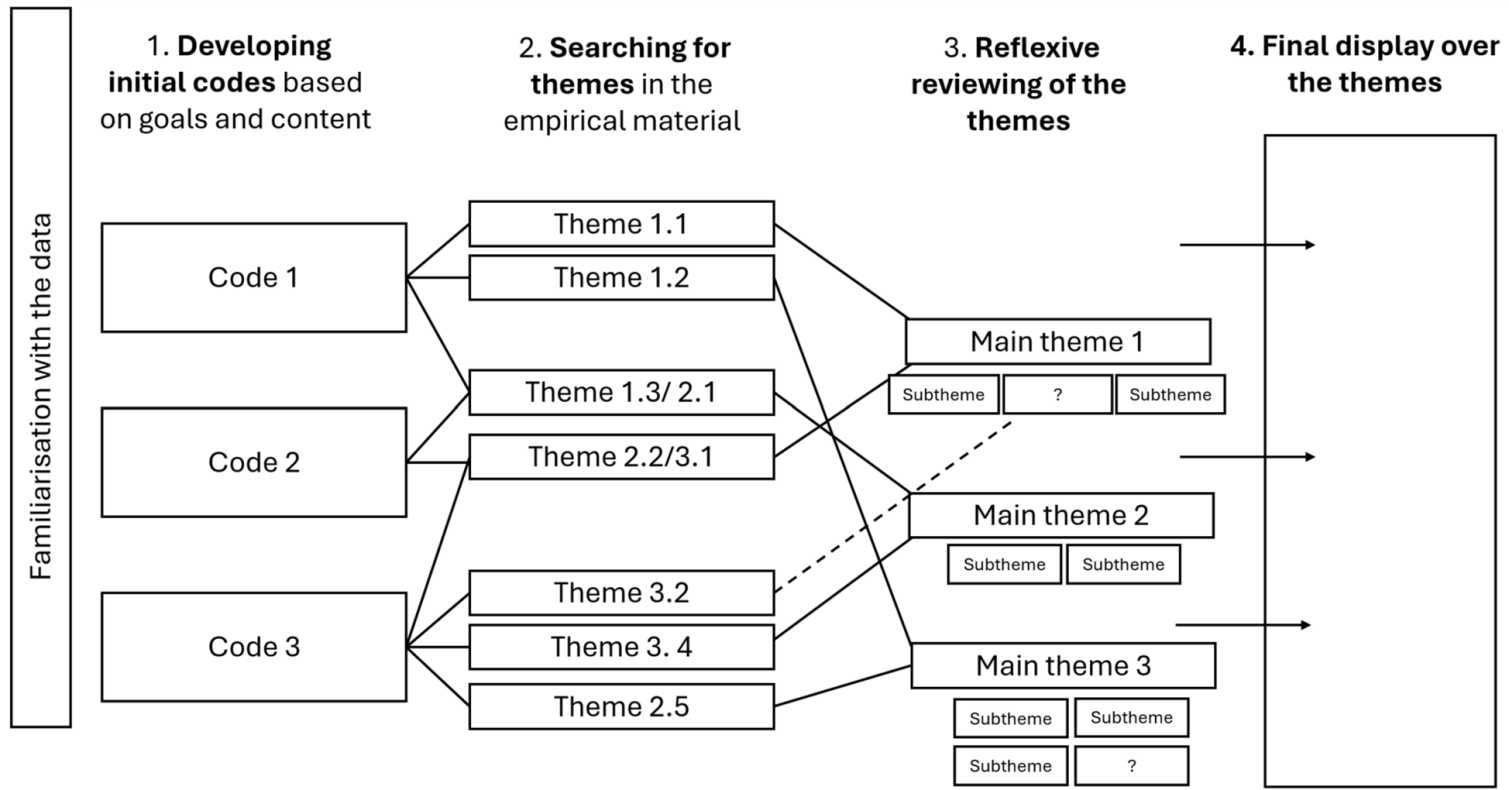
Steps in reflexive thematic analysis of qualitative data

Figure 6 illustrates the process of RTA of the data sets. The main steps distinguished by Clarke and Braun [116], such as familiarisation with the data, developing codes, as well as searching and reflexive revision of the themes, are included. This analytical strategy will lead to the final display of themes embracing the a) children’s experiences with the move-play-explore pedagogy, b) situated practices of movement-play-exploration in kindergartens and c) teachers’ meanings and experiences connected to implementation of the intervention. Elements from grounded theory will be employed to study children’s experiences (a) and situated practices (b), such as comparative approaches and deviant case analysis [215]. The main aim of grounded theory is to provide a systematic means for constructing theories of causation [216]. This approach allows for both inductive and deductive development of concepts by confirming or disproving hypotheses [217]. By using grounded theory, researchers can thoroughly familiarise themselves with and gain a profound understanding of the data. This deep comprehension is facilitated through the process of open coding, which involves researchers identifying common themes from various sources and agents [218]. To study implementation of the intervention (c) we will include testing of context-mechanism-outcome configurations developed prior to initiating the trial [199].

##### Children’s drawings

Analyses of children’s drawings will build on the assumption of drawings being “graphic representations” [219] (p. 77) of diverse experiences and events in children’s life [220, 221]. We hypothesize that the move-play-explore pedagogy will have an influence on the content of the children’s drawings. The 5–6-year-old children will be asked to draw: “In kindergarten, what was the most joyful activity you participated in this week?”. The content analysis of the drawings will register both the occurring themes and its frequency [222], which will be compared between intervention and control kindergartens. Each picture will be scanned and marked with group, the child age and gender. Next, the pictures will be scanned and coded in In vivo (Atlas. TI 8.0 software) where specific names will be given to individual elements pictured. These codes will be clustered thematically, and each of the pictures will be secondary coded as *including* or *not including* the identified themes. This dataset will be analysed quantitatively and compared between groups.

##### Quantitative data

In sample 1, we will analyse between-group effects on an individual and institutional level. In sample 3, we will analyse between-group effects or main effects of time among intervention kindergartens on an institutional level. Analyses will follow the approaches and procedures outlined in Sect. "Development of measures of movement competence, creativity, playfulness, explorative behaviour and well-being in young children" but will additionally apply Generalized Estimating Equations to model categorical data from questionnaires [223]. Analyses of potential moderators or mediators will be performed as described inSect. "Secondary aims", using both process and outcome evaluation data on the child and kindergarten level.

## Privacy protection and ethical perspectives

Our research group has extensive experience with large-scale data collections in children aged 3–6 years. All tests are specifically developed for young children and both physical and app-based measures are based on playful activities, which children generally enjoy. We will arrange all assessments in the kindergarten setting in collaboration with staff to meet each individual child’s needs of a safe environment and support. We acknowledge that assessments could cause stigma to children and families in the margins, children with disabilities, or children who grow up in families with little attention to ‘healthy living’. Efforts will be made to respect all children and families where normality can find many variations.

ECE is a gendered field with an almost entirely female workforce [224]. Regarding PA level and rough-and-tumble play, boys benefit more from the kindergarten setting than girls [20]. Moreover, it has been argued that traditional assessments of motor competence are biased towards skills integral to traditional male sports [225]. We will address these challenges by ensuring that gender perspectives are a key consideration in the development of the pedagogical design, assessment tools, and intervention implementation. We will examine moderation by gender to investigate a potential gender effect. The focus of MoveEarly may appeal to prospective and current male ECE staff, potentially contributing to improving gender equality in the ECE workforce [224].

The project will conform to the ethical guidelines defined by the World Medical Association’s Declaration of Helsinki and its subsequent revisions and has been approved by the Western Norway University of Applied Sciences’ ethics committee (reference number 22/11664–5). The study is conducted in accordance with Western Norway University of Applied Sciences’s internal control system and General Data Protection Regulation (EU 2016/679), approved by the Western Norway University of Applied Sciences’ privacy protection office and registered in the Sikt registry (reference number 186698). We will obtain written informed consent from each participant prior to data collection. We will follow the Vancouver recommendations for authorship policy to maintain high standards in scientific publishing.

## Data management and quality control measures

High quality in data collection, data management, and data analysis is of crucial importance to ensure valid study results and secure privacy protection of participants. Data collectors will be thoroughly trained prior to testing. A standardized protocol including a thorough description, pictures and/or videoclips of all assessments will be developed and used for training. Intertester reliability will be assessed for measures requiring observation. Training also includes a risk and vulnerability analysis as well as an introduction to the data management plan. This plan will ensure that scientific research data generated from the project will be easily discoverable, accessible, verifiable, useable for further research, and interoperable with existing standards. It is mandatory for all researchers who participate in data collection and data management in the MoveEarly project to comply with this protocol manual describing all procedures as approved by Western Norway University of Applied Sciences and documented in our Data Protection Impact Assessment. All non-digital data will be plotted and manually double-checked according to journal sheets and other sources of data, before the research file will be checked for illogical and unexpected values. After the thorough quality control of data, the database will be locked prior to opening the group allocation (18-month follow-up) and prior to performing any statistical analysis of intervention effects.

### Data sharing

The primary investigator and researchers in the MoveEarly project group will have access to the final datasets. The datasets generated in this project may be of interest to researchers, students, clinicians and policymakers within several fields. Sharing and re-use of data facilitate sustainability by increasing knowledge transfer, collaboration and reducing researcher and participant burden. We will obtain consent from participants to share and publish de-identified data to facilitate transparency and re-use of data. Data may be shared with internal and external parties by proposals directed to the primary investigator. As the data will not be anonymous, data will not be made openly available but will be available to researchers on reasonable request under agreed terms and conditions. Information about the project can be found on Western Norway University of Applied Sciences’ web page (https://www.hvl.no/en/research/project/moveearly/) and at ClinicalTrials.gov (https://www.clinicaltrials.gov/study/NCT06488508?cond=NCT06488508&rank=1). All publications related to the project will include contact information and scientific publications will include a DOI number. Table 1 provides metadata for the MoveEarly cluster RCT dataset. Table 1 .

**Table 1:** Metadata about the MoveEarly cluster RCT dataset

Key characteristic	Description
Data collection period	01.09.2024–30.06.2026
Timepoints	3 (Sept-Oct 2024, Apr-Jun 2025, Apr-Jun 2026)
Sample	Children aged 4–6 years
Sample size	Approximately 500 children
Type of data	Dataset
Software program	SPSS
Format	.sav
Storage	SILAF (internal secure research server)
Consent	Written informed consent by parent/caregiver
Sharing	A de-identified dataset will be available upon reasonable request
Data on vulnerable groups	Yes, children aged 4–6 years
Classified information	No
Transfer	Sikt Filesender, https://sikt.no/tjenester/filesender
Open repository	Not until the dataset is fully anonymized

## Interdisciplinary knowledge transfer

MoveEarly brings together several research and practice disciplines, paradigms, and theoretical approaches underpinned by distinct ontological and epistemological perspectives. While merging these disciplines can be challenging [94], it provides opportunities to foster diverse transdisciplinary collaboration to advance knowledge creation and transfer. Beyond scientific publications, we will focus on several priority groups, including ECE practitioners, education policymakers and public health policy makers, and employ comprehensive communication strategies to maximize knowledge transfer, guided by knowledge-to-action frameworks [226] and groups that our researchers are affiliated with. With few evaluated professional development programs available for the ECE sector [52], our ultimate goal if MoveEarly is successful – and with adaptations according to the process evaluation – is sustained dissemination of the intervention. We will offer the professional development model as part of the university in-service teacher training programs and include it in the existing undergraduate education programs. Thus, we will serve the kindergarten sector directly through our educational provision. Importantly, it will be feasible to increase the scalability of the intervention by structuring the professional development within a 15-credit university model that can be adapted by others. We will make the e-learning resource https://moveearly.no/ publicly available after the project is completed, to support teacher education, professional development and the kindergartens over the long-term. Collectively, this means that the distance from model testing to widespread dissemination is short, which we regard as a major strength for scaling the intervention and impacting public health and pedagogy in the early years.

## Discussion

Through MoveEarly, we will address the current absence of high-quality experimental evidence on how to develop children’s health, development, learning, and well-being through movement, play, and exploration. We hope to encourage researchers and ECE practitioners to move beyond siloed approaches aimed at obesity prevention or improving academic performance to holistic early years pedagogical approaches broadly targeting children’s needs and development. If successful, the project will show how ECE research can focus embodied and embedded approaches to learning. However, we have no guarantee for success, as there are challenges to implementation and institutionalization of the suggested pedagogy in kindergartens. By taking a pragmatic intervention approach, our premise of success is that we can increase kindergarten staff’s competence and motivation to include more high-quality movement, play and exploration in their everyday practices. Given limited time and workforce challenges in the ECE sector in Norway [7], this mission is not straight-forward. Yet, we regard the co-creation of the professional development with kindergarten staff, ensuring their autonomy and ownership, and the extensive professional development with 18-month follow-up from researchers significant strengths of the study, which increase the probability of success beyond many previous studies including less collaboration, teacher training and support [22, 23, 25, 26].

Beyond undertaking professional development and implementing the proposed pedagogical principles, a sufficient contrast in practices between intervention and control kindergartens is required to find any effects on a child level. Given that the concepts of movement, play and exploration are covered in the curricula for kindergartens in Norway [96], all core elements of the pedagogy is present in both intervention and control kindergartens. Since we are not providing a standardized “programme” for kindergartens to implement but “pedagogical principles” to guide their practice along with the tools and support, the contrast between groups is hard to predict. Evidence suggest both approaches (i.e., prescribed programs or guiding principles) may be equally effective [26]. Another prerequisite to reveal effects is the ability of outcome measures to capture potential intervention effects. While it is a strength of the study that we include a tailormade measure of movement competence and creativity and a range of commonly applied measures of health and development applicable for young children, we have a broad intervention and generic measures of child health and development. This approach contrasts, for example, proof-of-concept studies showing favourable effects of physically active learning on closely related learning outcomes [227, 228]. However, we aim to design an enriched pedagogical approach that ECE practitioners find meaningful and applicable and that easily can be adopted by kindergartens as a universal early years pedagogy. While it may be challenging to deliver the intervention both on a kindergarten (i.e., staff professional development) and child level (i.e., pedagogical practice), we argue that testing such an approach is critical to develop quality practices and inform policy development. Adding to the outcome evaluation, we include a thorough process evaluation to capture to which extent the intervention is implemented and institutionalized in kindergartens and how, under which conditions and for whom it may work [73]. This is critical to aid interpretation of our findings and improve knowledge of implementing complex interventions in the ECE sector and beyond.

We propose MoveEarly as a potential solution to shift societies toward a more active, healthy, and sustainable way of life. If successful, the project can have major benefits to society, by providing an important part of the puzzle to promote life-long PA, health, learning, well-being, agency, and life opportunities for the generations to come, thus supporting the UN Sustainable Development Goals on good health and well-being, quality education, and reduced inequality [229]. If the unified move-play-explore pedagogy is accepted by the ECE sector, it could have profound implications for policy development and uptake in the sector across local, regional, national, and international levels. As called for [52], of importance for dissemination and scaling, our intervention is framed within an education module that may be included in ECE educational programs and thus be available for the ECE workforce. However, scaling will likely be restricted to countries having a well-functioning kindergarten sector and educational systems.

## Supplementary Information

Supplementary Material 1.

## Data Availability

No datasets were generated or analysed during the current study.

## Abbreviations

ACTNOW: Active Learning Norwegian Preschool(er)s
BMI: Body mass index
CFA: Confirmatory factor analysis
CONSORT: Consolidated Standards Of Reporting Trials
CPM: Counts per minute
ECE: Early childhood education
EFA: Exploratory factor analysis
HTKS-R: Head-Toes-Knees-Shoulders Revised
ICC: Intra-class correlation
LPA: Light physical activity
MPA: Moderate physical activity
MVPA: Moderate-to-vigorous physical activity
PA: Physical activity
PREFIT: Assessing Fitness in Preschoolers
PRISMA: Preferred Reporting Items for Systematic reviews and Meta-Analysis
PRISMA-ScR: Preferred Reporting Items for Systematic reviews and Meta-Analysis extension for Scoping Reviews
RCT: Randomized controlled trial
RTA: Reflexive thematic analysis
SDT: Self-determination theory
SDQ: Strengths and Difficulties Questionnaire
SED: Sedentary time
SEM: Structural equation modelling
SPIRIT: Standard Protocol Items: Recommendations for Interventional Trials
SPSS: Statistical Package for the Social Sciences
TIDieR: Template for Intervention Description and Replication
VPA: Vigorous physical activity
